# Acquisition strategies for spatially resolved magnetic resonance detection of hyperpolarized nuclei

**DOI:** 10.1007/s10334-019-00807-6

**Published:** 2019-12-06

**Authors:** Geoffrey J. Topping, Christian Hundshammer, Luca Nagel, Martin Grashei, Maximilian Aigner, Jason G. Skinner, Rolf F. Schulte, Franz Schilling

**Affiliations:** 1 Department of Nuclear Medicine, Klinikum rechts der Isar, Technical University of Munich, Munich, Germany; 2 General Electric Healthcare, Munich, Germany

**Keywords:** Magnetic resonance imaging (MRI), Hyperpolarization, Spectroscopy, Spectroscopic imaging (MRSI), Metabolic imaging

## Abstract

Hyperpolarization is an emerging method in magnetic resonance imaging that allows nuclear spin polarization of gases or liquids to be temporarily enhanced by up to five or six orders of magnitude at clinically relevant field strengths and administered at high concentration to a subject at the time of measurement. This transient gain in signal has enabled the non-invasive detection and imaging of gas ventilation and diffusion in the lungs, perfusion in blood vessels and tissues, and metabolic conversion in cells, animals, and patients. The rapid development of this method is based on advances in polarizer technology, the availability of suitable probe isotopes and molecules, improved MRI hardware and pulse sequence development. Acquisition strategies for hyperpolarized nuclei are not yet standardized and are set up individually at most sites depending on the specific requirements of the probe, the object of interest, and the MRI hardware. This review provides a detailed introduction to spatially resolved detection of hyperpolarized nuclei and summarizes novel and previously established acquisition strategies for different key areas of application.

## Introduction

The main limiting factor in molecular imaging by magnetic resonance imaging (MRI) is its inherently low sensitivity, resulting from low spin polarization at thermal equilibrium and exacerbated by low concentrations in vivo of many compounds of potential interest that contain NMR-active isotopes. These limitations can be overcome temporarily by increasing the polarization by more than five orders of magnitude beyond thermal equilibrium, which is possible through several hyperpolarization techniques [1], and administering the exogenous hyperpolarized substance to the subject during or shortly before acquisition. The most prominent techniques, dissolution dynamic nuclear polarization (dDNP) [2] and spin exchange optical pumping (SEOP) [3], have led to breakthroughs in molecular imaging by means of MRI. Detection of biologically relevant hyperpolarized ^13^C-labeled substrates in the micromolar concentration range, such as [1-^13^C]pyruvate [4], ^13^C-bicarbonate [5], or [1,4-^13^C_2_]fumarate [6], as well as their respective metabolic products [1-^13^C]lactate, [^13^C]carbon dioxide, and [1,4-^13^C_2_]malate, has become possible, and can give new insights into biochemical pathways in vivo [7]. The evaluation of the clinical potential is currently underway, with more than 20 ongoing clinical trials focusing on hyperpolarized [1-^13^C]pyruvate [8]. Imaging of inhaled hyperpolarized noble gases ^129^Xe [9] and ^3^He [10] with SEOP and its application for measurement of flow [11] and diffusion [12] is used clinically primarily to assess lung health [13–16], particularly in the acinar airways that cannot be assessed with computed tomography [17].

Several different techniques have been developed to overcome the low net spin polarization imposed by the Boltzmann distribution at thermal equilibrium at clinically relevant field strengths. By various means, these methods induce a transient state of increased nuclear polarization that decays with the spin–lattice relaxation time *T*_1_. Five different approaches are currently used to create a hyperpolarized spin state for various nuclear isotopes, which currently include ^1^H (protons), and various others (X-nuclei): ^3^He, ^6^Li, ^13^C, ^15^N, ^29^Si, ^31^P, ^129^Xe, ^83^Kr, and ^107,109^Ag:

1. Brute-force hyperpolarization methods that exploit low temperatures and high *B*_0_ field are generally applicable but achieve only moderate polarization levels [18, 19].
2. Spin exchange optical pumping (SEOP) is used to polarize the noble gases ^3^He [10], ^83^Kr [20], and ^129^Xe [9] using circularly polarized laser light to excite specific transitions of alkali metal vapors. The polarization is then transferred to the nuclei of the noble gas atoms by spin exchange collisions of the alkali metal and the noble gas atoms [3]. The hyperpolarized gas may be delivered and measured as a gas or after dissolution in a liquid solvent [21].
3. Parahydrogen-induced polarization (PHIP) uses a chemical reaction to transfer the high para-state spin order of hydrogen to bonded atoms through *J*-coupling [22] in the liquid state, e.g. for ^13^C [23].
4. Signal amplification by reversible exchange (SABRE) transfers polarization from parahydrogen to the molecule of interest in the liquid state using an activated catalyst for, e.g. ^1^H, ^13^C, ^15^N, [24, 25], and ^31^P [26, 27].
5. Dissolution dynamic nuclear polarization (dDNP) can transfer electron polarization to nuclear spins in the solid state at cryogenic temperatures, which are subsequently heated, dissolved in a liquid, and preserved at room temperature, e.g. for ^13^C [2], ^6^Li [28], ^15^N [29], ^19^F [30], ^29^Si [31], ^31^P [32], and ^107,109^Ag [33].

Regardless of hyperpolarization technique and route of administration into the subject or object of interest, efficient acquisition strategies are needed, which are adapted to the properties of hyperpolarized signals. The hyperpolarized magnetization decays with *T*_1_, which can be on the order of a few tens of seconds in vivo (e.g. for ^13^C compounds [34, 35] or ^129^Xe in an MR magnet [9]). Each RF excitation further reduces the available signal for subsequent excitations, which is particularly limiting when using dDNP, which typically provides single doses of hyperpolarized compound solution with intervals of more than 30 min between dissolutions. This is also the case for inhaled gas imaging, in which the available magnetization (and thus signal) can be replenished at most once per subject breath. Hyperpolarized compounds and their metabolites, particularly those labeled with ^13^C or ^15^N, as well as ^129^Xe in various solutions or trapped in molecular cages [36–38], can cover a wide range of chemical shifts, which can hinder or help the design of sequences to separate their signal contributions, particularly in combination with the smaller gyromagnetic ratio of non-proton nuclei and the consequent need for higher gradient field strengths. For metabolic imaging, it is often necessary to acquire temporally resolved data, in order to extract metabolite dynamics. For imaging of lung ventilation, temporally resolved data allow gas flow to be visualized [11]. Temporally resolved data can also help ensure that the peak signal is measured after a hyperpolarized compound is administered, as the timing of its arrival in the area of interest may not be known prior to the measurement.

In this article, we review acquisition strategies for the measurement of hyperpolarized nuclei that are used to address these challenges, including strategies for pre-scan adjustments, hardware and field strength considerations, and sequence components including spectral encoding, spatial encoding, and excitation and contrast. We also discuss in more detail several important acquisition strategies and individually notable pulse sequences, including non-imaging spectroscopy, free induction decay (FID) chemical shift imaging (FID-CSI), echo planar spectroscopic imaging (EPSI), spiral multi-echo methods, free precession sequences, spectral-spatial excitation, and relaxometry.

## Hardware considerations

In addition to the pulse sequence, system hardware, including most notably the static magnetic field strength and the radiofrequency (RF) coils used, are important considerations for localized hyperpolarized spectroscopy and imaging experiments.

### Static magnetic field strength

The static magnetic field (*B*_0_) strength has several impacts on hyperpolarized nuclear magnetic resonance imaging and spectroscopy, with some differences to those of thermal proton measurements.

The imaging system field strength does not determine polarization level [39] or strongly affect signal strength for hyperpolarized compounds that are introduced from an external source [40]. Hyperpolarized measurements have thus been performed in vivo at 3 mT with ^3^He [40, 41], 15 mT with ^129^Xe and ^3^He [42], 48.7 mT with ^13^C [43], and 100 mT with ^3^He [39], which are much lower field strengths than are typically used for thermal proton measurements.

There are several advantages associated with lower static magnetic field strengths for hyperpolarized measurements. Lower field strengths show reduced susceptibility effects, resulting in threefold increase in *T*_2_* at 0.43 T compared with 1.5 T for ^3^He imaging in vivo, providing an effective increase in signal strength for sequences with long echo times [44]. The *T*_1_ relaxation rates of many hyperpolarized ^13^C compounds are also strongly dependent on the *B*_0_ field strength [45]. Lower field strengths can also compensate for the greater demands placed on gradient systems with X-nuclei due to their lower gyromagnetic ratio than that of protons. The same *k*-space trajectory, slice profile, or pulsed-gradient diffusion weighting will require proportionally larger gradient strengths for X-nuclei. This can be particularly problematic for sequences that traverse *k*-space rapidly with long readout gradients, such as multi-gradient echo spectroscopic measurements of ^13^C compounds, which can display a much wider range of chemical shifts [46] than protons in most biologically interesting compounds, and thus benefit from rapid echo spacing and associated wide spectral bandwidth. At lower fields, however, these gradient demands are proportionally reduced. Additionally, heteronuclear decoupling of protons, particularly for molecules containing ^13^C or ^15^N atoms, can require prohibitively large RF power in humans at high field for decoupling large spectral bandwidths due to specific absorption rate (SAR) restrictions [47, 48]. Last, lower field permanent magnets can be advantageous due to their lower costs compared with cryogenically cooled superconducting magnets [39].

As with thermal proton spectroscopy, a major advantage of higher field strengths for hyperpolarized spectroscopy is improved separation of spectral peaks [47, 49]. This is useful with hyperpolarized nuclei in liquids, such as precise measurement of chemical shifts for ^13^C [50] or frequency-selective saturation with ^129^Xe HYPER-CEST [38]. Additionally, peak splitting due to *J*-coupling, affecting molecules containing multiple spin ½ atoms, such as protons and ^13^C or ^15^N, is not field dependent, and is thus less problematic at higher field strengths, without the need for decoupling.

Hyperpolarized spectroscopy and imaging measurements in vivo are normally conducted in combination with thermal proton imaging for anatomical reference to guide the planning of the hyperpolarized measurement, to provide complementary parametric or functional information, and for pre-scan adjustments. As such, the most suitable field strength for hyperpolarized imaging is often the field of an existing MRI system, to which X-nucleus imaging capability is added. The impacts of field strength on proton imaging are thus also relevant for most hyperpolarized X-nucleus experiments.

### Radiofrequency coils

Radiofrequency (RF) coils and resonators are used to transmit and receive the radiofrequency fields that both manipulate magnetization and convey the signal in NMR and MRI measurements. For both proton and X-nucleus measurements, RF coils are produced in a wide variety of sizes and configurations, designed for particular measurement scenarios and geometries. RF coils may be categorized in by three independent properties: (1) the *B*_0_ field, nucleus, and frequency for which an RF coil is resonant, (2) the coil size and shape, including whether coils are volume resonators or surface coils, and (3) whether coils are single channel, multi-channel, or phased arrays. These properties directly impact the types of hyperpolarized measurements for which a coil is suitable.

RF coils are designed to be resonant within a relatively narrow band of frequencies, in order to be optimally sensitive within that band and be minimally sensitive to other signals. For hyperpolarized measurements on standard NMR and MRI systems, which are normally designed and used primarily at proton frequencies, additional RF coils designed for X-nucleus applications are required. For X-nuclei with substantially lower gyromagnetic ratio than that of protons (i.e. those other than ^19^F), the Larmor frequency at which the RF coil operates is similarly reduced, which affects its noise characteristics [10, 51]. For example, at 3 T, 128 MHz for ^1^H is sample noise dominated, whereas 32 MHz for ^13^C has relatively more noise contribution from the coil [52], meaning improved coil design can be more impactful. Dual resonance coils are also available for ^1^H and ^13^C, ^23^Na, or ^31^P, [48, 53–58], which are useful for heteronuclear ^1^H-^13^C decoupling [48, 59] and polarization transfer applications, which involve simultaneous transmission on multiple frequency channels. Triple resonance coils [60, 61] can also be helpful for *B*_1_ calibration of hyperpolarized compounds containing ^13^C by using signal from natural abundance ^23^Na, because their Larmor frequencies are similar.

Coil geometry substantially impacts the applications for which the coil is suitable. Volume resonators surround the subject and provide relatively uniform *B*_1_ transmit and receive profiles, and are most suitable for imaging applications where the overall distribution of signal across the field of view is important, such as when the signal magnitude in well-separated regions is directly compared, e.g. for lung gas distribution [16, 62]. Even when a surface receive coil is used, a separate volume coil is often used for transmission, due to the importance in some pulse sequences of uniform and well-calibrated transmit *B*_1_, which can be particularly challenging to achieve with hyperpolarized measurements. Surface coils, conversely, produce strongly spatially varying transmit and receive *B*_*1*_ profiles [63], dependent on the distance from and size of the coil [64], which can reduce noise and amplify signal near the coil. This can be an advantage when a superficial tissue or organ is being studied, such as preclinical studies of subcutaneous tumors [50, 65–67], brain [68, 69], heart [70–75], liver [37, 64], or kidneys [50], and clinical studies of the heart [76]. However, spatially varying intensity from surface receive coils can substantially complicate interpretation of images, even when taking ratios of multiple metabolite signals. Additionally, endorectal coils have been developed for human prostate hyperpolarized ^13^C imaging [77–80].

Quadrature coils [60, 81, 82] provide a theoretical √2 increase in *B*_1_ over similar-geometry linear coils, but provide no independent receive or transmit channel capability. Multiple independent channel coil arrays enable pulse sequence optimizations for hyperpolarized measurements, which are not possible with single-channel linear and quadrature coils. Receiver arrays coils with 4, or 8 channels have been used for hyperpolarized ^13^C parallel imaging [83–85], including controlled aliasing for chemical shift separation [86]. An array with 32 channels has been used for hyperpolarized ^129^Xe parallel imaging [87]. Multi-channel arrays are also advantageous for improved spatial coverage with good SNR in hyperpolarized imaging [88].

Cryogenically cooled coils can improve SNR through reduction of coil noise compared to coils operating at room temperature [51]. This can be particularly helpful for X-nuclei imaging, with lower Larmor frequencies than that of protons, as coil noise has a larger impact on SNR [52]. A threefold SNR enhancement has recently been demonstrated for a 30 × 40 mm^2^^13^C cryocoil at 3 T [89].

## Pre-scan adjustments

Before MRI measurements, several pre-scan adjustments may be performed, including shimming the static magnetic field [90], sequence *k*-space trajectory measurement, and transmit *B*_1_ calibration. Although some gas imaging uses exogenous hyperpolarized gas for pre-scan adjustments, for most liquid-state hyperpolarized magnetization is non-renewable, so pre-scan adjustments are not usually done with the same hyperpolarized magnetization that is used for subsequent measurement. Instead, because all nuclei are influenced by the same *B*_0_ field, shimming for X-nuclei is most often performed using signal from protons [60] in the subject, with shim current adjusted iteratively [90] or based on measured *B*_0_ field maps [91, 92]. *k*-space trajectories can similarly be measured with proton signal [49, 93, 94], with correction for the different gyromagnetic ratios between nuclei [95], or can be measured with a highly concentrated thermally polarized X-nuclei phantom. Techniques for transmit *B*_1_ calibration are discussed below.

### *B*_1_ calibration for X-nuclei

Transmit *B*_1_ calibration is necessary to determine accurate RF pulse amplitudes, which are necessary in many sequences to produce the desired contrast, optimal signal, and artifact-free images [96]. For lower-frequency coils with stable coil loading, transmit *B*_1_ calibration may be performed just once, and the same calibration used for all subsequent measurements. In other cases, calibration may be repeated separately for each new subject.

Sequences with higher flip angle pulses are in general more sensitive to flip angle accuracy, including non-adiabatic inversions and refocusing pulses to produce spin echoes [97]. Flip angle accuracy is also important for the quantification of metabolite concentrations [96]. Sequences with low flip angles, especially sequences with a series of variable (and low) flip angle pulses [98], as well as adiabatic RF pulses [99], are less affected by the *B*_1_ calibration.

For thermal proton MRI, several standard transmit *B*_1_ calibration procedures are used on preclinical and clinical MRI systems [100–104], using signal from the subject. For X-nuclei, however, transmit *B*_1_ calibration is more challenging. Due to their low in vivo concentrations and smaller gyromagnetic ratio, natural abundance X-nuclei often produce too little signal to be used for thermally polarized *B*_1_ calibration [105]. Furthermore, most hyperpolarized signal is non-renewable, and is thus often not suitable for use in *B*_1_ calibration [73], with the exceptions of ^3^He and ^129^Xe lung imaging [62, 87]. Instead, a separate high concentration gas or liquid thermally polarized phantom is normally used to calibrate *B*_1_ before most X-nuclei hyperpolarized measurements [106–108]. For coils with subject-dependent loading, such a phantom may be placed adjacent to the subject, near the region or structure of interest to ensure similar coil loading and local *B*_1_, and may be removed or left in place for the subsequent hyperpolarized measurement. Alternatively, a phantom may be designed to simulate the presence of a subject [105].

Some pulse sequences additionally require receive coil *B*_1_ profiles for each independent channel of multiple-channel coils, independent of the transmit coil *B*_1_ profile. These include parallel reconstruction with sensitivity encoding (SENSE) [83, 109, 110]. Receiver coil *B*_1_ calibration has most of the same limitations for hyperpolarizing imaging applications, relating to lack of signal for calibration measurements.

Several methods have been developed for calibration of the transmit *B*_1_ field in measurements using hyperpolarized X-nuclei. Some methods produce only a single *B*_1_ calibration factor for a given experimental configuration, which may only be accurate within a limited spatial region of the subject, particularly for surface transmit RF coils or subjects that extend outside a volume coil. Other methods produce a *B*_1_ map, which indicates the spatial variation of flip angles.

#### Pulse power incrementation

A thermally polarized phantom is placed next to the subject, and the non-selective or slice-selective FID signal is measured after excitation at varying RF power levels (or nominal flip angles). The resulting curve can be fitted with a sinusoid [111], or the flip angle calibration determined from the point where the spectral peak crosses zero (or its phase inverts) [107], which corresponds to a 180° excitation pulse [105, 112] at the location of the phantom. To produce an accurate calibration, the phantom must be limited in size and placed in a location with the same local *B*_1_ as the region of interest in the subject.

#### Scaling *B*_1_ for different nuclei

The *B*_1_ transmit calibration for X-nuclei can be estimated using the *B*_1_ calibration of another nucleus with a similar gyromagnetic ratio and Larmor frequency. This proportionality will be different for any unique pair of coils, however, and will not account for local *B*_1_ variations. The *B*_1_ calibrations of ^1^H and ^3^He have been shown to be proportional between separate single-nucleus volume coils [113]. The *B*_1_ calibration for ^13^C can also be estimated with that of ^23^Na using a dual-tuned ^23^Na/^13^C coil with a scaling factor, which is useful because ^23^Na is present in most biological tissue at relatively large concentration, which simplifies the design and operation of such coils [96].

#### Repeated excitation with small flip angle

A series of RF excitation pulses or spoiled gradient echo images with constant power can be used to measure the *B*_1_ calibration or generate *B*_1_ maps from the signal variation with number of excitations [114, 115]. This is similar to the variable power method, except that it requires approximate initial estimate of the *B*_1_ calibration and *T*_1_ relaxation time, but uses only small flip angles. The spacing between excitation pulses may also be varied to simultaneously measure *T*_1_ and the *B*_1_ calibration [116].

#### Magnetization inversion

Flip angle measurement by magnetization inversion can be applied to hyperpolarized gas. Magnetization is prepared with two RF excitation pulses, separated by a *B*_0_ field gradient to produce a spatially varying phase. The RF pulses have flip angle above 45°, so that magnetization is inverted (more than 90° effective flip angle) after both pulses, in a band where the gradient field is weakest. The flip angle of the RF pulses can then be determined from the width of the inverted band of magnetization, and is unaffected by oxygen-related relaxation, imperfect slice profiles, or diffusion [114].

#### Bloch–Siegert shift

**Fig. 1:**
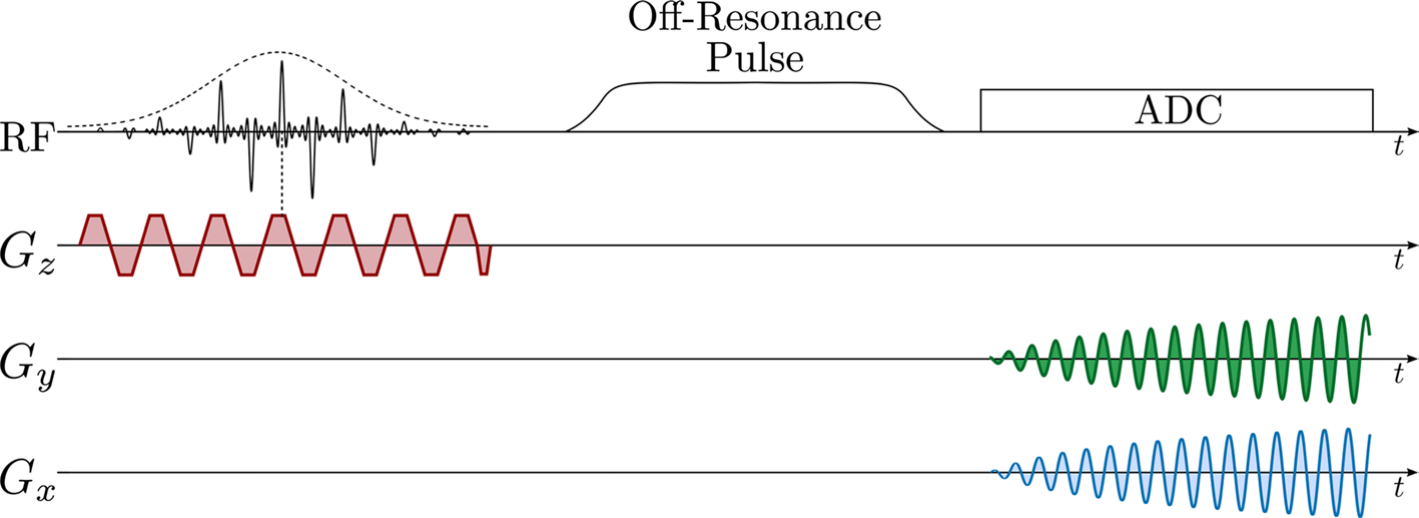
Bloch–Siegert *B*_1_ mapping sequence diagram, with spectral-spatial RF pulse, off-resonance RF pulse, and spiral readout gradients

**Fig. 2:**
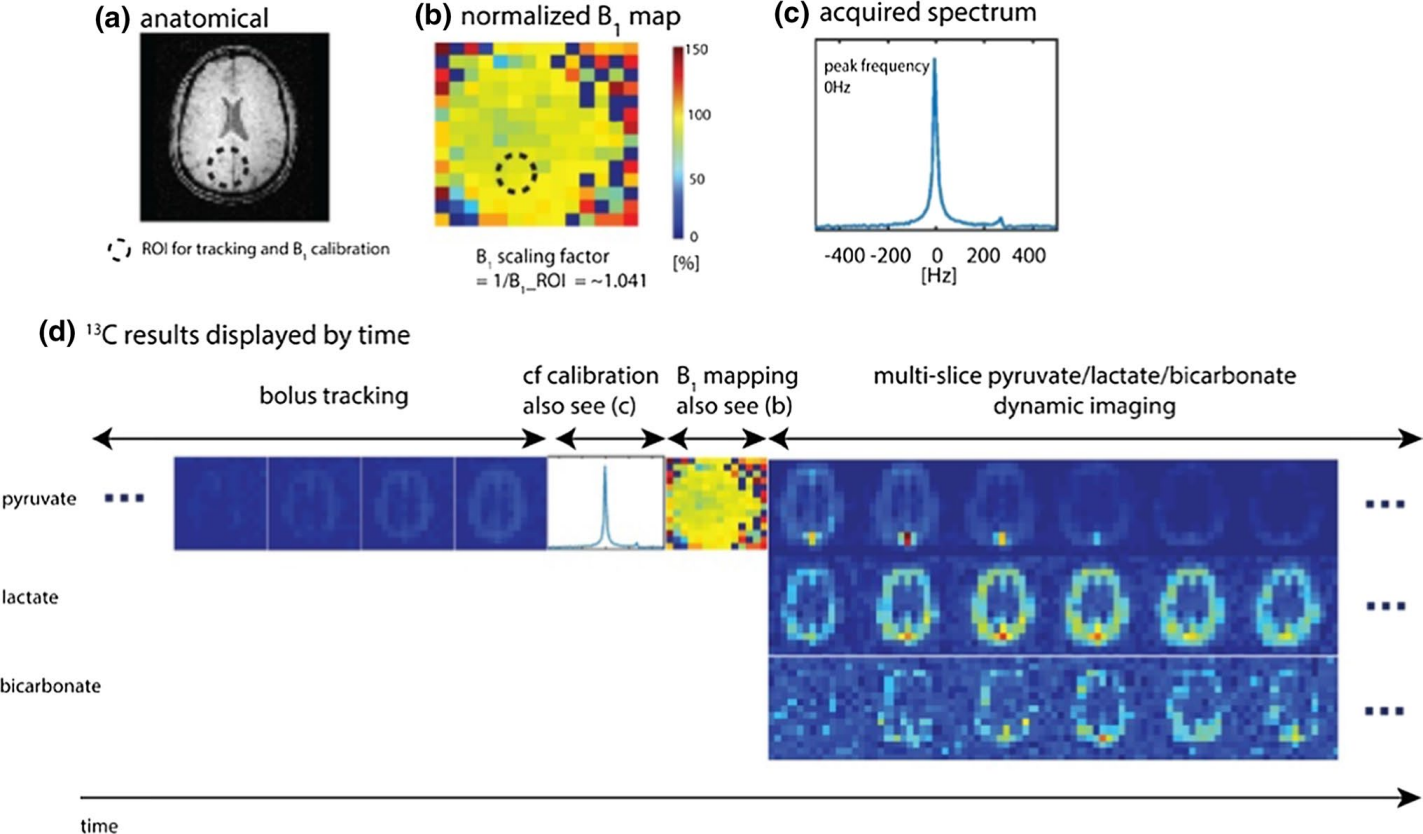
Results of hyperpolarized [1-^13^C]pyruvate studies on a healthy human volunteer using bolus tracking, Bloch–Siegert *B*_1_ mapping, and multi-slice dynamic imaging of ^13^C metabolites. **a** Proton image of slice 5. **b** Normalized ^13^C *B*_1_ maps. **c** Acquired frequency spectrum for center frequency calibration. **d**^13^C results of slice 5 displayed in the order of time. Reprinted with permission from Tang et al. [119]

The Bloch–Siegert method is a relatively fast method for *B*_1_ calibration and measuring *B*_1_ maps [117]. Magnetization is excited, and then an off-resonant RF pulse is applied to produce a *B*_1_-dependent phase shift [118]. An example pulse sequence for mapping *B*_1_ using this method is shown in Fig. 1. The Bloch–Siegert method has been used for slice-selective *B*_1_ calibration with thermal ^13^C phantoms and hyperpolarized ^3^He in lungs [112], and for *B*_*1*_ mapping during dynamic in vivo ^13^C-pyruvate image acquisition [73] and triggered by bolus tracking immediately before dynamic in vivo ^13^C-pyruvate image acquisition (Fig. 2) [119].

#### Signal reduction

When two low-flip angle images or spectra are acquired in succession, of non-renewable magnetization that does not flow into or out of the excited volume, the second image’s intensity is reduced due to excitation losses and *T*_1_ relaxation. This has been applied for flip angle mapping in human lungs, with breath holding, of hyperpolarized ^129^Xe and ^3^He [62, 87]. Similar methods are also used for in vitro spectroscopic measurements [120].

## Pulse sequence components

A wide variety of pulse sequences have been developed for measuring hyperpolarized nuclei, from simple NMR acquisitions to sophisticated optimized sequences. These sequences in general use some combination of (1) spectral encoding, (2) spatial encoding, and (3) excitation and contrast scheme. Various possible pulse sequence elements are listed below, within these categories. Not all variations can or have been combined, but each usable sequence will use one or more of the methods in each category.

### Spectral encoding

Spectral encoding methods separate NMR signal by frequency (chemical shift) of the nucleus being measured. Different chemical shifts of the same nucleus arise from different local magnetic fields at the sites of the nucleus, which are induced by the different chemical environments in different molecular species that incorporate the nucleus, by their different positions within a single molecular species, or the solution, surface [121], or molecular cage [38] in which it resides. In cases with only one measurable chemical shift, no spectral encoding is required. If more than one distinct chemical shift is present, spectral encoding isolates the signals at those frequencies, and thereby measures their distinct amplitudes, distributions, or time-courses. This separation may be accomplished with frequency selective excitation, during signal readout from the phase evolution of a time-series of measurements, or a combination of these two methods.

Most measurements of hyperpolarized agents have relatively sparse spectra. Only one or a small number of distinct molecules are usually present in the administered hyperpolarized agent. These molecules may be chemically stable during the time course of measurement, may be rapidly metabolized into a small number of additional compounds, or may be present in a small number of distinguishable chemical environments. Furthermore, the design of a hyperpolarized experiment will usually ensure that the compounds of interest have relatively well-separated chemical shifts. This is in contrast to many in vivo proton NMR spectroscopic measurements, in which many endogenous compounds appear close together in the spectra. For such hyperpolarized signals with relatively easy to separate sparse spectra, a variety of spectral encoding schemes can be used.

#### Free induction decay (FID)

Signal is measured without applied gradients [4, 34, 122–126]. The spectrum is the Fourier transform of the FID signal. This method can provide wide spectral bandwidth without placing high demands on the gradient hardware.

#### Multi-echo model-free

Multiple gradient echoes are acquired with evenly spaced echo times [127, 128]. The echo train at each *k*-space point may be treated similarly to an FID [129], depending whether the echoes are acquired with symmetric or flyback gradient shapes. The spacing of echoes should be sufficiently short to give spectral bandwidth that is adequate to separate the frequency offsets of the compounds of interest, without problematic frequency wrapping of the spectrum [130].

#### Multi-echo sparse

A relatively small number of gradient echoes are acquired [131]. At least one more echo than distinct spectral components is needed [132], and the number and timing of echoes may be adjusted to optimize sensitivity to and separation of an expected number and distribution of frequency components in the signal [133]. Images for expected frequency components are separately reconstructed from the phases of measured echoes and the known timing of the echoes using matrix inversion [125, 134] or iterative least-squares (IDEAL) [132, 135] methods.

#### Chemical shift offset separation

Images are acquired with frequency encoding during readout with sufficiently low receive bandwidth that multiple frequency components in the signal are chemically shifted to produce separate images [136], or partially aliased images that can be separated with parallel imaging reconstruction [86].

#### Frequency selective excitation

Frequency selective RF pulses excite or invert a single frequency, a narrow range of frequencies [137], or alternate between frequencies [138].

#### Spectral-spatial (SPSP) excitation

Specially designed RF pulses, in combination with spatial encoding gradients, are used to excite within a band of frequencies that include only a single hyperpolarized compound and within a spatially limited region [65, 139] or which selectively excite multiple frequency bands [140, 141]. Multiple alternating excitations also can be used to acquire images of multiple frequencies [70].

#### Spatiotemporal encoding

RF excitation or inversion pulses are swept in frequency (chirped or adiabatic) while an encoding gradient is active, and a decoding gradient is applied during signal readout [142, 143]. Spatially resolved spectral content of the resulting signals can be separated [142, 144].

#### No spectral encoding

Images are acquired with a non-spectroscopic MRI sequence. This method is used for hyperpolarized ^129^Xe imaging [9, 106, 145], ^3^He imaging [146–148], or other angiography imaging [34, 149, 150] for which spectroscopic separation of multiple chemical shifts is not needed.

### Spatial encoding

Spatial encoding separates NMR signal by location in space. Most in vivo measurements require some form of spatial encoding in the pulse sequence or receive coil(s) to localize signal to an organ or other region of interest or to produce images that are spatially resolved in two or three dimensions. Conversely, most in vitro measurements use the total signal from the entire sensitive volume of the receiver coil, with no additional spatial localization than that from the limited size of the object and the coil’s sensitivity profile.

NMR signal spatial localization may be accomplished with the sensitivity profiles of receiver coils, spatially selective RF pulses, field gradients after excitation and before or during signal acquisition, or with a combination of these methods.

For imaging hyperpolarized agents with non-renewable magnetization, fast spatial encoding is required, as is keeping the number of RF excitations used to acquire the image data minimal. This is particularly true for multi-frame acquisitions, which need relatively short times between frames to follow signal dynamics, and also need to preserve magnetization for later frames. Various trajectories and strategies for traversing *k*-space have been used for hyperpolarized imaging, mainly initially for thermal proton imaging and subsequently adapted. These include several techniques for accelerating the acquisition by under-sampling data in the spatial and spectral dimensions and recovering the missing information using parallel receiver coil arrays or by taking advantage of the sparsity of the spectral dimension.

#### Hardware-sensitivity localized

The receiver and transmit radiofrequency (RF) coil hardware sensitivity profiles (*B*_1_ field) determine the measurement’s spatial sensitivity to the signal. The pulse sequence itself does not localize the origin of the signal [34].

#### Slice selective excitation

The excitation radio frequency (RF) pulse is applied while a linear gradient field is active [151, 152]. The excited volume is a thin 2D slice or thick 3D slab in space [65]. If more than one chemical shift is present in the object [121, 153], there is a spatial offset between the slices corresponding to different chemical shifts [68]. This offset leads to the so-called chemical shift displacement artifact, which can be reduced by using a high transmit bandwidth relative to the chemical shift offset, or avoided with spectral-spatial excitation pulses.

#### Voxel-selective excitation

Three orthogonal slice-selective excitation and refocusing or inversion RF pulses restrict the excited volume to a cuboidal voxel [154, 155]. The resulting signal may be spin or stimulated echoes, depending on the pulse phase and amplitude. Alternatively, slice-selective outer volume suppression [156, 157] may be applied prior to excitation, to the volumes surrounding a target volume. Outer volume suppression does not require echo generation using multiple slice-selective high-flip-angle excitation, refocusing, or inversion pulses applied to the target volume.

#### Phase encoding

Phase-encoding gradient blips are applied after excitation and before readout [122, 124] or repeatedly during readout [158, 159] to move between *k*-space points or lines.

#### Frequency-encoded readout

Frequency encoding gradients are active and constant during signal readout [9]. This is typically combined with an initial dephasing blip, during phase-encoding, so that the phase of the image passes through the center of *k*-space in the frequency encoding direction during the readout, producing a gradient-recalled echo.

#### Multi-gradient echo

A train of gradient echoes is acquired to read multiple *k*-space lines [146] (EPI) or multiple echoes of the same line [128, 160, 161] (EPSI) per excitation or refocusing RF pulse. *B*_0_ field inhomogeneity can limit the use of long echo trains [12] or require correction algorithms [162].

#### Symmetric

Frequency encoding gradients during readout have alternating polarity, moving through *k*-space in opposite directions [134], also referred to as bipolar readout gradients. Chemical shift offsets and inconsistency in gradient response between directions can lead to differences between odd and even numbered echoes [163], for which bipolar gradient corrections have been developed [164].

#### Flyback

Frequency encoding gradients are constant during readout, alternating with gradient pulses that use the maximum slew rate to rewind the encoding and prepare for the next readout [165, 166]. Compared with symmetric or non-frequency-encoded readouts, encoding efficiency is lower, as data are not acquired during rewind pulses.

#### Cartesian *k*-space trajectory

*K*-space points are acquired in a standard MRI pattern of orthogonal rows and columns (or also a third dimension). Grid spacing may be uniform or uneven depending on whether constant or ramped gradients are used during acquisition.

#### Centric encoding

*K*-space points or lines are acquired in a center-out order [4, 147], rather than sequentially across *k*-space. This improves signal strength with non-renewable hyperpolarized magnetization and is less sensitive to motion, but can increase spatial blurring artifacts [167].

#### Spiral *k*-space trajectory

Gradients are continuously adjusted during readout to trace a spiral pattern from the center to the periphery of *k*-space. *K*-space may be covered in a single spiral, giving non-spectroscopic data [11, 70], or a series of spirals giving spectroscopic data [125, 131, 168]. Such a pattern will produce a non-Cartesian sampling of *k*-space, and thus will require a suitable reconstruction method [131, 169, 170].

#### Radial trajectory

Frequency encoding is applied radially out from the center of *k*-space [123]. This can give very short effective echo times for gas *T*_2_* measurement, or useful oversampling of the center of *k*-space, giving good sensitivity to contrast changes for dynamic imaging [171–174]. Alternating radial gradients to produce multiple echoes have also been used for ^13^C-pyruvate spectroscopic imaging [175].

#### Concentric rings trajectory

Frequency encoding is applied in a series of retraced circles [176, 177]. This trajectory has timing, sampling efficiency, and gradient-demand advantages compared with spiral or Cartesian *k*-space sampling trajectories.

#### Under-sampling

Image data are under-sampled in the spatial and spectral dimensions during acquisition and the reconstruction compensates for the missing information. Pseudo-random under-sampling is referred to as compressed sensing [79, 158, 159, 178–181]. Regular under-sampling, with fully sampled training data in the center of *k*-space, can be reconstructed with *k*-*t* principal component analysis, using a limited number of temporal basis functions that are derived and used to constrain the image reconstruction for each spatial location [74, 182, 183].

#### Parallel imaging

A multi-receiver coil array is used during acquisition with a field of view that does not fully cover the subject [86]. Reduced field of view, and thus spatial aliasing of the image, is equivalent to omitting *k*-space lines during acquisition, which is corrected in reconstruction using information from the spatial distribution of the receiver array. Parallel reconstruction methods include sensitivity encoding (SENSE) [83, 109, 110], autocalibrating parallel acquisition (GRAPPA) [12, 87, 184], and calibrationless parallel imaging [185–187]. Omitting *k*-space lines allows fewer excitations [87] and higher flip angles [12] to be used in sequences using multiple excitations, improving temporal resolution without loss of SNR, as would be the case with thermal polarization [188], or reducing overall scan time [12]. Alternatively, multiple slices are excited simultaneously, and signals are separated using coil sensitivity information [189].

#### Partial Fourier

Symmetry in *k*-space is exploited to under-sample by omitting acquisition of almost half of the *k*-space [84, 148, 190].

#### Spatiotemporal encoding

RF excitation or inversion pulses are swept in frequency (chirped or adiabatic) while an encoding gradient is active, and a decoding gradient is applied during signal readout [142, 143, 191]. The excited magnetization has a phase that varies quadratically in space, and the decoding gradient moves the vertex of the phase parabola, where magnetization is in phase, during readout [192].

### Excitation scheme and contrast

RF pulses and static magnetic field gradients are used to manipulate magnetization to produce signal and to control its contrast and sensitivity to various physical properties in the subject. Every NMR measurement has an excitation pulse, which tilts magnetization into the transverse plane, where its precession produces the RF signal that is acquired. Further RF and *B*_0_ field gradient manipulations may be applied prior to excitation, between excitation and readout, or during readout.

For hyperpolarized agents, non-renewable magnetization limits sequence timing and the use of contrast mechanisms that are common for thermally polarized measurements. Flip-back RF pulses and balanced gradients are of increased importance, rather than spoiling residual transverse magnetization after readout. For refocusing and inversion, adiabatic RF pulses are preferred due to their relative insensitivity to transmit power calibration, as are non-spatially selective pulses, to avoid mixing of inverted and non-inverted magnetization and saturation effects adjacent to the targeted slice at high flip angles. Sequence repetition times are kept to a minimum, to avoid loss of hyperpolarized magnetization due to relaxation, compared with thermally polarized acquisition, in which longer repetition times allow relaxation to increase the longitudinal magnetization available for subsequent excitations. For hyperpolarized gases, diffusion imposes further limits on sequence design.

#### Constant flip angle

Magnetization is excited at a constant flip angle RF pulse [9, 124].

#### Variable flip angle

Flip angle is varied between excitations or chemical shift. Angle may be increased with time to compensate for loss of magnetization due to previous excitations and *T*_1_ decay [25, 130, 193–195]. The flip angle may also be varied between frequency-specific excitations, when one compound has much higher concentration than another, or to avoid depleting the substrate of a reaction being investigated (e.g. pyruvate being converted to lactate) [65, 140, 162, 178]. Angle may also be varied in a more complicated pattern over time and chemical shift to optimize estimate precision of a rate constant [196].

#### Spin echo

Magnetization is excited and then refocused with a second RF pulse or train of refocusing pulses [149]. Spin echoes are useful for hyperpolarized gases which have short *T*_2_* due to diffusion effects [146, 149], unless the sequence also involves intense gradient activity [145]. Spin echo sequences can also rapidly deplete hyperpolarized magnetization [145] and are sensitive to flip angle calibration [146]. Inverted magnetization will also mix with non-inverted magnetization through blood perfusion, resulting in opposite-polarity cancellation and loss of signal [128].

#### Adiabatic refocusing

Magnetization is excited at a low flip angle and then refocused by a pair or train of adiabatic inversion [197] spatially non-selective RF pulses [140]. Adiabatic pulses are relatively insensitive to flip angle calibration and off-resonance effects [198], and can produce good refocusing and low signal loss from dephasing [199]. By refocusing twice, the magnetization is effectively returned to the state it had after the initial low flip angle excitation, allowing an effective low flip angle to be used, preserving magnetization for subsequent acquisitions. A train of adiabatic refocusing pulses can produce a series of spin echoes, with signal acquired after each refocusing pulse [199]. Blood perfusion magnetization cancellation issues are also avoided by inverting non-selectively [128].

#### Free precession

Magnetization is repeatedly and rapidly (relative to the relaxation times *T*_1_ and *T*_2_) excited [200] and refocused with constant [34, 150] or variable flip angles [195], with alternating phase, and without spoiling gradients. Typically, balanced gradients are used, which have zeroth gradient moment of zero between successive RF pulses [34, 134, 201–204]. Free precession sequences are often referred to as (balanced) steady-state free precession (bSSFP), although for hyperpolarized measurements, no measurable steady state is reached, due to the lack of magnetization recovery from *T*_1_ relaxation, as occurs in thermal proton measurements.

#### Stimulated echo

Magnetization is excited and tilted back into the longitudinal direction after a short dephasing time. The prepared magnetization is then excited by a third RF pulse after a mixing time. This produces a stimulated echo [205] with timing dependent on the timing of the RF pulses [206]. Stimulated echoes can be used to provide diffusion or perfusion weighting [207, 208] for hyperpolarized ^13^C imaging. Stimulated echoes can be preferable to spin echoes for measurements using long echo times [209].

#### Saturation and inversion

Using a frequency-selective RF pulse, magnetization is saturated or inverted [138]. This can be useful to observe how metabolite signals exchange after selective saturation of ^13^C-labeled molecules [66], to investigate how selectively saturated magnetization of encapsulated ^129^Xe chemically exchanges with the hyperpolarized ^129^Xe pool in solution at a different chemical shift (HYPER-CEST) in [38], or to selectively destroy signal outside a region of interest to ensure that metabolites flowing into a region do not disturb the temporal relationship between metabolites [210]. Saturation is also used in continuous flow (replenished) hyperpolarized ^129^Xe gas measurements of microstructural exchange properties in materials [211] and lungs [13].

#### Polarization transfer

Polarization transfers from one nucleus to another spontaneously due to heteronuclear cross-relaxation [212], or the transfer may be induced by RF pulses [213], such as with (reverse) insensitive nuclei enhanced by polarization transfer (INEPT) [35, 157, 214–216]. Hyperpolarizing ^13^C or ^15^N and then transferring the polarization to ^1^H before measurement is useful due to their longer *T*_1_ relaxation times [217, 218] and because nuclei with higher gyromagnetic ratios place lower demands on the gradient system for equivalent signal encoding. Furthermore, assuming efficient transfer of polarization and minimal losses due to other pulse sequence differences compared with direct X-nucleus polarization measurement, the higher gyromagnetic ratio (*γ*) of protons theoretically provides higher detection sensitivity [35, 46, 215] and SNR, proportional to *γ*^2^.

#### Diffusion

Apparent diffusion coefficient (ADC) may be assessed with paired pulsed gradients [219, 220]. Varying strength and separation of the gradient pulses controls the diffusion weighting *b*-value and thus the amount of motion-dependent dephasing and signal intensity reduction [221]. Diffusion over longer distances can also be measured with spatial modulation of the longitudinal magnetization [222]. In vivo hyperpolarized diffusion imaging is used to assess lung microstructure [12, 223] with gases and liver fibrosis [224] with ^13^C. Most imaging sequences will also have some inherent diffusion weighting, dependent on the strength of gradients they use [145, 148].

#### Flow

Sensitivity to liquid flow or perfusion can be introduced with stimulated echo pulse sequences, which dephase magnetization before the second RF pulse, and rephase it after the third RF pulse unless the magnetization has moved from its initial position [207, 208, 225]. Phase variation in vitro has also been used to image flowing hyperpolarized water [226].

#### Static/single or dynamic/multiple excitations

A single image is acquired [4, 9], or multiple images are acquired over time, allowing a time-course of signal to be reconstructed [65, 146, 227].

#### Spoiling

After signal readout, substantial magnetization may remain in the transverse plane, although it will usually have been dephased by phase and frequency encoding. The transverse magnetization may be further dephased (spoiled), by again turning on a strong gradient [131, 150], so that it will not contribute directly to the signal after subsequent RF pulses or produce stimulated echoes.

#### Decoupling

NMR spectral peaks can split due to heteronuclear *J*-couplings. Particularly at lower field strengths, this leads to separation of signal into multiple peaks, which may be independently less distinguishable from background noise. Continuous wave RF irradiation at the Larmor frequency of a nucleus, e.g. proton, that is coupled to an X-nucleus, e.g. ^13^C, suppresses the *J*-coupling evolution during the signal acquisition and removes the peak splitting. This has been used on clinical MRI scanners with hyperpolarized compounds, and produced narrower spectral line widths [228] and substantial sensitivity improvement [64] in vivo, and in non-imaging NMR measurements of hyperpolarized compounds [229]. In addition, off-resonance decoupling has been used to obtain heteronuclear chemical shift correlations from hyperpolarized compounds [230–232].

## Pulse sequences

Many pulse sequence variations have been proposed and demonstrated for hyperpolarized imaging and spectroscopy, which make use of various combinations of the sequence components discussed above. Several important sequences or groups of sequences are discussed below, in rough order from less to more complicated, including non-imaging and imaging methods.

### Non-imaging spectroscopic methods

NMR spectroscopic methods omit spatial encoding gradients during signal acquisition and (in most techniques) during RF excitation. The signal may still be localized with the sensitivity profiles of the RF transmit and/or receiver coils, or with gradients applied during excitation and refocusing RF pulses to provide slice or voxel-selectivity. These methods employ a wide variety of contrasts and spectral encoding schemes for hyperpolarized experiments.

#### Non-localized spectroscopy

Non-localized spectroscopy originates with first nuclear magnetic resonance (NMR) experiments of Bloch [233] and Purcell [234]. RF pulses are used to invert, saturate, refocus, and excite magnetization across, and signal is received from, the entire sensitive volume of the RF transmit and receive coil. For hyperpolarized nuclei, the most common acquisition scheme is free induction decay (FID) [2, 39]. Single high-angle or multiple low-flip angle iterative excitations are applied, typically spaced several seconds apart [137] to follow physical, physiological, or metabolic processes for up to several minutes [124]. Constant or variable flip angle schemes are used [235]. Material science applications include Xe gas adsorption on solids [121]. In vitro experiments include enzymatic assays [236], cell metabolism experiments [65], or whole resected organs [237], at a wide range of field strengths from 0.0487 T [43] to 14.1 T [238]. Because the entire sample is contained within the sensitive volume of the coil, inflow and outflow of hyperpolarized magnetization do not need to be accounted for, facilitating kinetic modeling [239].

MRI systems are also used for non-localized in vivo spectroscopy [34, 124], when the subject fits within the RF coil. In any other case, signal will be localized by the sensitivity profile of the coil, even without any localization from the pulse sequence.

#### Localization by coil sensitivity

The *B*_1_ field profile of a surface coil [63, 65] can be exploited to isolate signal near the coil’s position. A small solenoid or volume coil [227] can similarly be placed over a region of interest to isolate signal within. Both the RF transmission and reception *B*_1_ profiles can be used, with transmission to limit the volume of excited magnetization, and with reception to limit the volume from which signal is received. When used to transmit, surface coils produce spatially varying effective excitation angle (less so with adiabatic pulses) which can complicate signal interpretation. Signal localization by coil sensitivity profile can also be combined with selective excitation and spatial encoding with magnetic field gradients.

In vivo, surface receive coils are used for detection of signal from superficial regions and near-surface organs such as subcutaneously implanted tumors [21], heart, liver, or head [240]. Coil size is adapted to the application, from more than 13 cm diameter [241], down to 8 mm [242], and most commonly 20 mm in preclinical small animal experiments [75, 243, 244].

#### Slice-selective excitation

Slice selective or spectral-spatial pulses are used to excite magnetization in a slice, limited in one spatial dimension. Typical slice thicknesses for hyperpolarized experiments range from 5 mm [65] to 40 mm [245].

As with non-selective excitation, slice selective excitation can be applied iteratively at low flip angles, preserving hyperpolarized magnetization during dynamic measurements. For in vivo studies, however, in-flowing magnetization can complicate quantitative modeling. Multiple slices may be interleaved with alternating excitations, in order to simultaneously measure separate body regions [153] or objects [246]. Slice excitation can also be combined with surface-coil localization [65]. This approach is well suited for single organs [25, 243] or subcutaneous tumors [65].

#### Voxel-selective (echo) spectroscopy

An excitation pulse is followed by orthogonal-slice selective RF pulses to produce a spin or stimulated echo within a cuboidal voxel. Stimulated echo acquisition mode (STEAM) [247, 248] produces a stimulated echo with three 90° pulses. Point resolved spectroscopy (PRESS) [155, 249] produces a spin echo with an excitation pulse followed by two 180° refocusing pulses. Localization by adiabatic selective refocusing (LASER) [197] produces a spin echo with a non-selective excitation and three pairs of adiabatic refocusing pulses.

Both STEAM and PRESS, using shaped slice-selective pulses or spectral-spatial excitation, require accurate *B*_1_ transmit calibration to produce effective refocusing pulses. Adiabatic pulses are less sensitive to *B*_1_ calibration. Adiabatic and spectral-spatial pulses provide improved robustness to the chemical shift displacement artifact, which can complicate data interpretation [250] with simple slice selection. They can also produce low effective flip angles for dynamic acquisition, without saturation effects outside the target voxel, as can occur with high angle simple slice selective pulses [155]. These saturation effects can also be mitigated for single time-point sequential acquisition in multiple voxels by careful orientation and positioning of voxel edges to preserve magnetization in other regions [251]. Such multi-voxel approaches [252] can be an efficient alternative to imaging for isolating signal to a small number of discrete regions of interest [154].

#### Multi-dimensional and exchange spectroscopy

Two-dimensional NMR spectroscopy traditionally involves multiple excitations with varying parameters, such as evolution or mixing times between excitations or excitation frequencies [253].

Exchange spectroscopy (EXSY) employs stimulated echoes with varied evolution time between the first and second RF pulses and fixed mixing time between the second and third RF pulses [254]. During the evolution time, the magnetization acquires a phase at a rate dependent on its chemical shift, which is separable from the chemical shift measured during the readout, allowing a 2D spectrum to be extracted that describes the distribution of exchanging magnetizations before and after the mixing time. EXSY can be applied to renewably hyperpolarized magnetization in vitro, such as generated with SABRE [255].

For two-site exchange, MAD-STEAM [256, 257] can be applied with only a single encoding and acquisition, with slab selective excitation, making it more suitable for hyperpolarized measurements in vivo. MAD-STEAM produces a phase shift in magnetization that depends on the frequency difference between the two exchanging sites and the echo time. This technique allows signal of in-flowing metabolite to be separated from that arising from metabolic conversion. Ultra-fast exchange spectroscopy (UF-EXSY) [252] extends MAD-STEAM, with multiple echoes and sparse sampling to separate multiple exchanging metabolites and voxel selective excitation.

Ultrafast two-dimensional spectroscopy uses a series of frequency-stepped slice selective excitations [258–260] or swept-frequency excitations [261] or inversions [262] and a multi-echo readout. Although the excitations are localized, they are applied to a uniform object and are used to parallelize the evolution times. This method has been applied to hyperpolarized ^13^C and ^15^N in combination with polarization transfer to ^1^H before readout [46, 260, 263].

#### Diffusion spectroscopy

Diffusion weighting can be introduced with paired pulsed gradients with spin echo [219] or stimulated echo [209, 256] acquisition. For hyperpolarized measurements, because the magnetization decays rapidly and non-renewably, the mixing time [264] and echo time [265] are kept short and constant, with gradient strengths varied to control the diffusion weighting *b*-value, and higher diffusion weightings are acquired first to optimize SNR [221, 266]. Saturation recovery sequences can also produce diffusion weighting in continuous flow experiments [267].

Diffusion weighting is used to characterize in vivo microstructures [221, 268], transgene expression [67], and transport processes [269]. Hyperpolarized in vitro diffusion spectroscopy with ^13^C has been used to separate intra- and extra-cellular metabolites [264], in combination with non-diffusion-weighted spectra to separate signal decay from *T*_1_ or metabolic conversion [266].

### Free induction decay chemical shift imaging (FID-CSI)

**Fig. 3:**
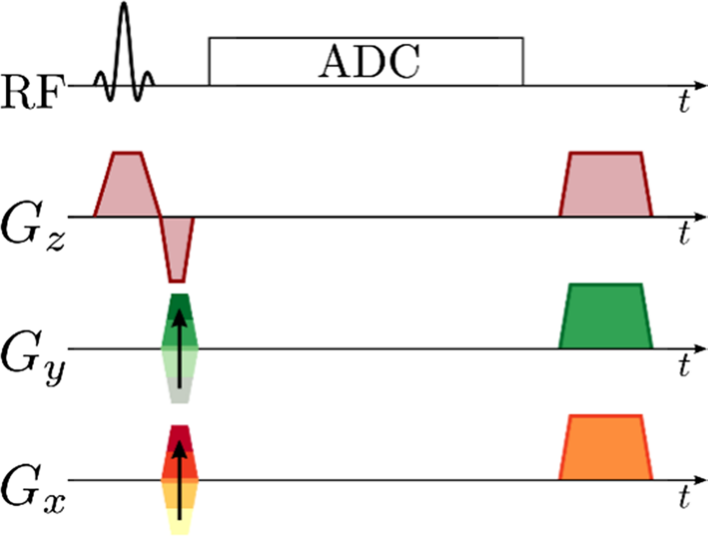
FID-CSI pulse sequence with slice-selective excitation and 2D phase encoding, acquisition window without any gradients active, and post-acquisition spoiler gradients

**Fig. 4:**
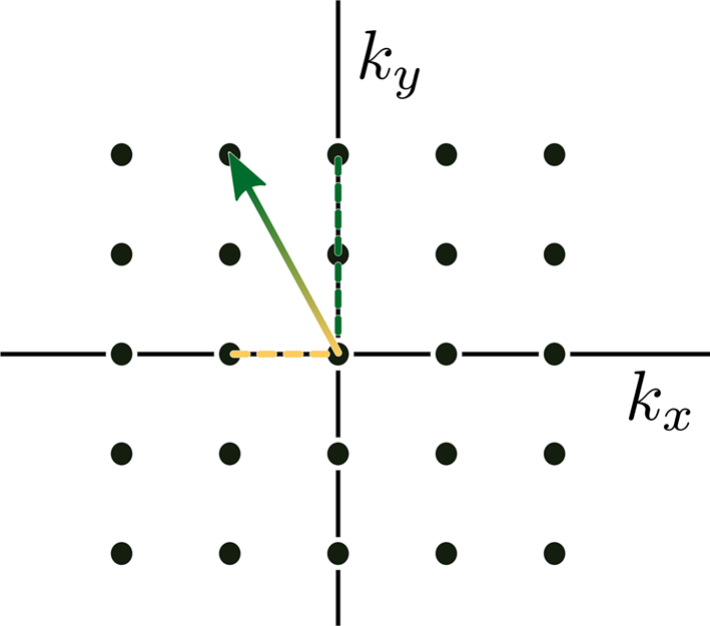
*k*-space acquisition pattern of a 2-spatial-dimensional FID-CSI pulse sequence. Phase encode gradients are applied simultaneously for all spatial dimensions before acquisition starts so that a single point in *k*-space is encoded for the duration of each acquisition. Subsequent acquisitions are used to acquire additional *k*-space points

Free induction decay (FID) chemical shift imaging (FID-CSI) is the simplest spectroscopic imaging method and was used early for spectroscopic imaging of hyperpolarized ^13^C compounds [4] and for ^129^Xe in gas and liquid phases [137]. It consists of 2D single-slice, multi-slice, or 3D slab selective low-flip angle excitation, 2D or 3D phase encoded spatial localization, and FID readout with no gradients active [122]. An example pulse sequence for mapping *B*_1_ using FID-CSI is shown in Fig. 3, and an example pattern of *k*-space acquisition is shown in Fig. 4.

Because FID-CSI does not use any gradients during readout, it places relatively little demand on the gradient system and is minimally affected by non-ideal gradient response and eddy currents. It is also relatively robust to static field (*B*_0_) inhomogeneity, which is particularly helpful at higher field strengths, and can be used to measure *B*_0_ variations from spectral frequency shifts from voxel to voxel in the image. Furthermore, FID-CSI is able to acquire with broad spectral bandwidths [270], limited by the digitizer sampling rate, rather than the rate at which gradient echoes can be generated as in many other sequence types.

In the context of hyperpolarized imaging, FID-CSI uses magnetization relatively inefficiently because each *k*-space point is separately read out as an FID after phase-encoding [270]. Maximizing spectral resolution requires a readout long enough for the transverse magnetization to have mostly decayed, which requires a long TR, which can make the time to acquire a full *k*-space prohibitively long [271], during which the longitudinal magnetization is also decaying. Shorter readout can be used, at the cost of limiting spectral resolution and wasting any remaining transverse magnetization, which is then typically spoiled before the next excitation. Even with short readout for each *k*-space point, spatial matrix sizes are limited [134]: typically 16x16 pixels for static dissolved ^129^Xe or ^13^C imaging [65, 124, 131, 271].

**Fig. 5:**
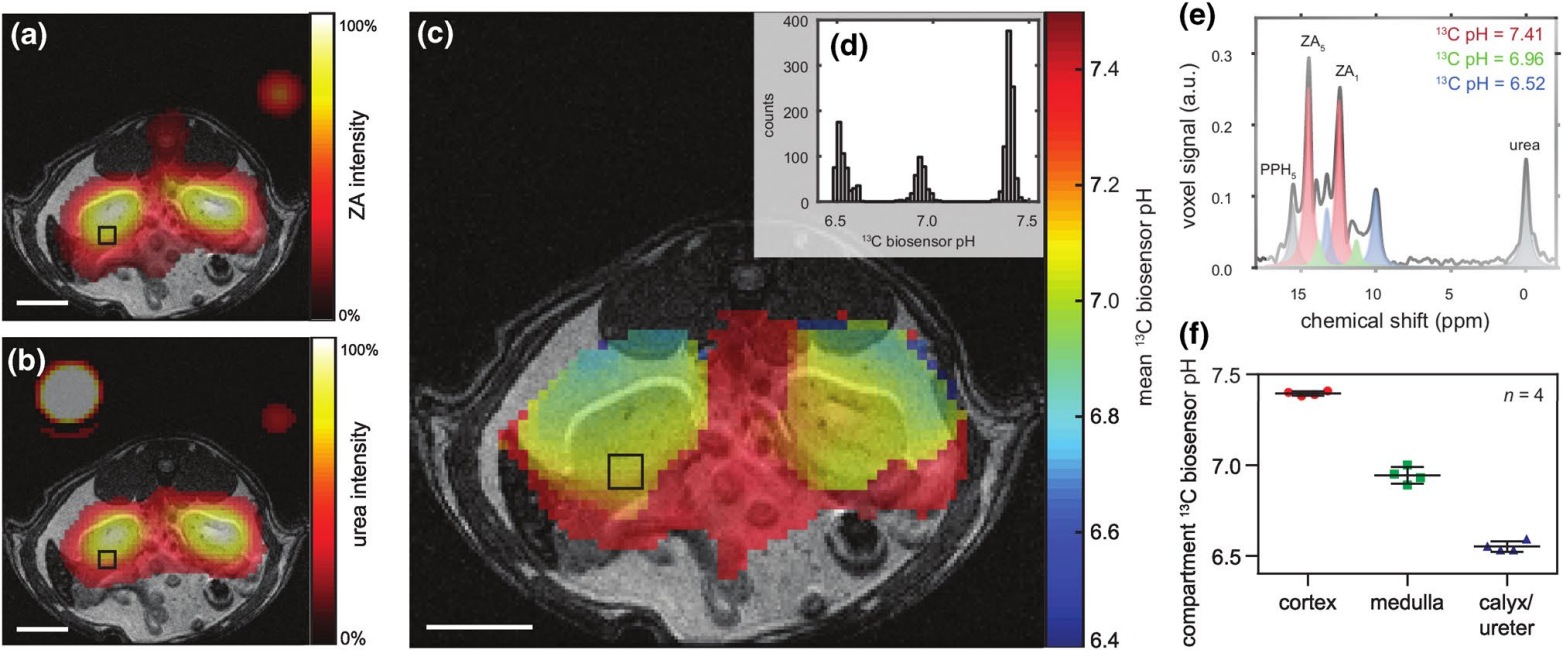
Hyperpolarized ZA in vivo pH measurements show three pH compartments in rat kidneys at 7 T. Representative kidney data from a hyperpolarized ^13^C measurement (colored) in an axial slice overlayed on anatomical proton images (grayscale). A calibration phantom containing ^13^C urea and the catheter used for injection are visible. The two simultaneously hyperpolarized and injected substances (**a**) ZA and (**b**) urea show high signal intensities in both kidneys of a healthy rat. (**c**) The mean pH map shows lower pH values in the kidneys compared to the surrounding tissue. (**e**) A voxel can contain up to three pairs of ZA peaks (red, green, blue) and a noticeable amount of PPH. The pH values group into three clusters (**d**, shown for one representative animal), consistently demonstrated in four animals (**f**, individual datapoints and mean ± s.d.). For all ^13^C images, intensity windows are based on sufficiently high signal levels for either (intensity images) or both (pH images) ZA and urea. Scale bars, 1 cm. Reprinted with permission from Düwel et al. [50]

FID-CSI is relatively robust; it can be used with compounds with multiple chemical shifts that are not precisely known prior to imaging (Fig. 5), and in cases with *B*_0_ variation, which leads to broader spectral line widths and variations in peak position across the field of view. Multi-frame FID-CSI is suitable when very limited spatial resolution and moderate spectral resolution are acceptable, and when the temporal resolution may be relatively poor (on the order of 5 s per time frame). Multi-slice FID-CSI is also possible, with additional compromises on the temporal, spectral, and spatial resolutions. 3D FID-CSI is generally impractical for multi-frame, as even a minimal 8 × 8 × 8 matrix will require too many phase-encodes and excitations per frame to have meaningful time resolution and dynamic time-course information with a hyperpolarized substance.

### Echo-planar spectroscopic imaging (EPSI)

**Fig. 6:**
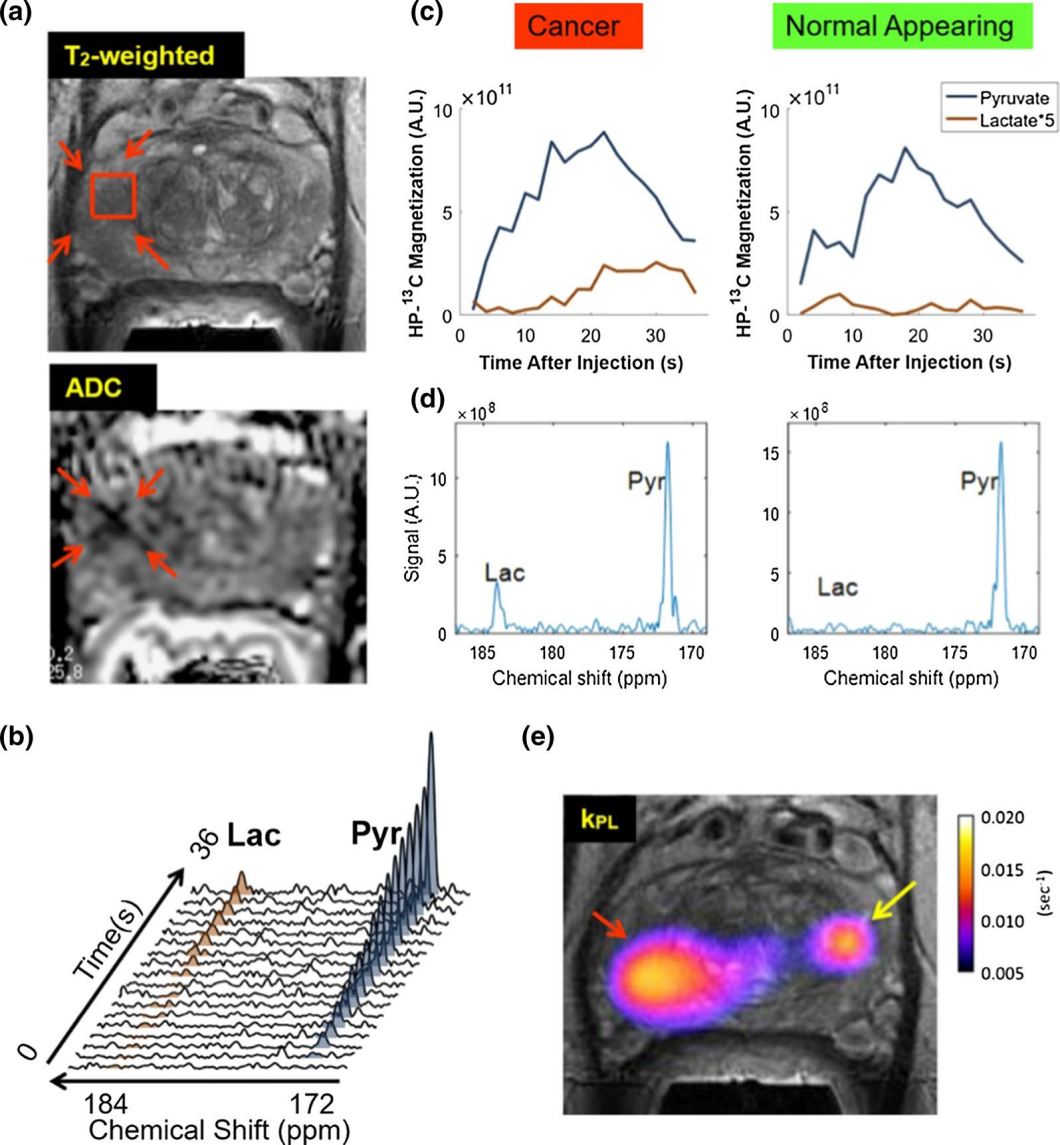
Dynamic echo-planar spectroscopic images (EPSI) of hyperpolarized ^13^C pyruvate to lactate conversion in clinical prostate tumor patient. **a** Anatomical and diffusion images. **b** Dynamic pyruvate and lactate time course within voxel indicated on anatomical image. **c** Flip-angle-corrected dynamic pyruvate and lactate time-courses in tumor and normal-appearing tissue. **d** Representative spectra at *t* = 36 s. **e** Pyruvate-to-lactate conversion *k*_PL_ parameter map, with localized high values marked with arrows, which were later confirmed as Gleason score 4 + 3 prostate cancer by post-surgical histopathology. Reprinted with permission from Chen et al. [79]

Echo-planar spectroscopic imaging (EPSI) is an accelerated spectroscopic imaging sequence and is the most commonly used sequence for clinical hyperpolarized [^13^C]pyruvate studies [272, 273]. It uses an oscillating readout gradient to sample one line in *k*-space multiple times after each excitation [160]. The spectral and one spatial dimension are thus sampled simultaneously. Additional spatial dimensions [158] are phase encoded between excitation and readout (Figs. 5, 6).

EPSI uses hyperpolarized magnetization relatively efficiently for spectroscopic imaging, in contrast with FID-CSI, because it acquires (the equivalent of) a full line of *k*-space after each excitation. Acquisition times can be on the order of a few seconds for fully sampled single images of hyperpolarized compounds. EPSI can thus be used for dynamic sampling of biochemical pathways [270] (Fig. 6) with ^13^C in tissues, but may be impractical for gas imaging due to long effective echo times and diffusion-related dephasing while gradients are oscillating.

Particularly for non-proton nuclei with lower gyromagnetic ratios, the gradient strength and slew rate determine the minimum echo spacing and thus the maximum spectral bandwidth [273], potentially limiting the metabolites that can be imaged without spectral aliasing with EPSI. Gradient strength also imposes limits on the minimum field of view and the maximum spatial resolution in the readout direction, which can be problematic for smaller subjects at higher static field strengths.

**Fig. 7:**
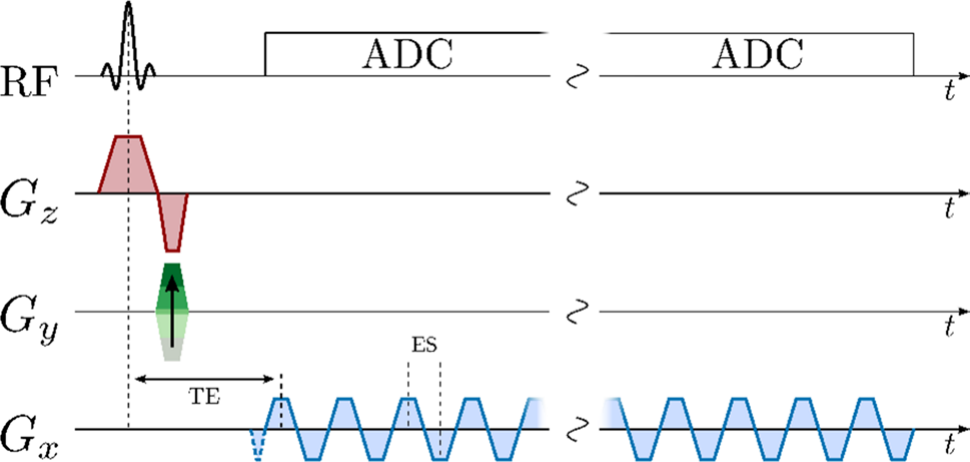
EPSI pulse sequence with slice-selective excitation pulse, a single phase-encoding gradient blip after excitation, and symmetric bipolar readout gradients moving in alternating directions through *k*-space during a multi-echo acquisition

**Fig. 8:**
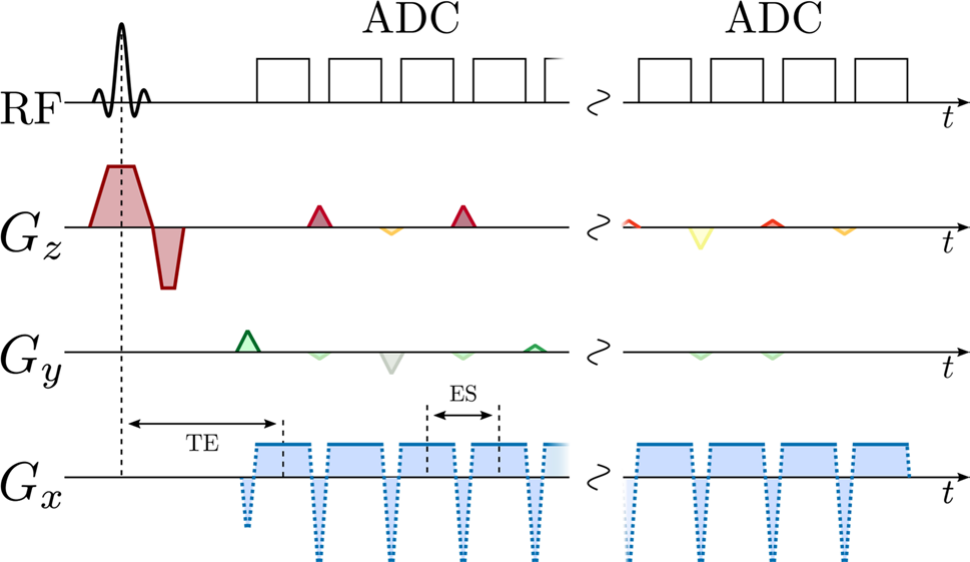
EPSI pulse sequence with slice-selective excitation pulse, flyback gradient pulses between signal acquisitions that all move in the same direction through *k*-space, and phase-encoding gradient blips in *k*_*y*_ and *k*_*z*_ between acquisitions, to move between *k*-space lines to accelerate acquisition by pseudo-randomly under-sampling the spectral and *k*-space information

**Fig. 9:**
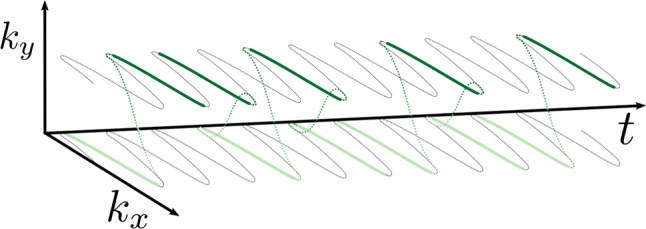
Model flyback EPSI *k*-space trajectory with just two phase-encodes. Pseudo-random phase-encoding jumps in *k*_*y*_ occur simultaneously with rapid flyback to low *k*_*x*_ (dotted green lines) between the signal acquisitions (solid green lines), which always move in the same direction through *k*-space, from low to high *k*_*x*_

Several *k*-space encoding strategies are used with EPSI. Symmetric readout gradients [273] acquire data while moving in alternating directions through *k*-space, minimizing echo spacing. However, this may lead to odd–even echo inconsistencies and spectral ghosting related to static field inhomogeneity [274] and gradient-induced eddy currents [275]. Ghosting may be reduced by separately reconstructing the odd and even echoes at the cost of a reduced spectral bandwidth [273]. Flyback readout gradients acquire data in only one gradient readout direction [128, 166] and eliminate odd-even echo inconsistencies, but at the cost of higher echo spacing, lower spectral bandwidth [273], and lower sampling efficiency [95] because data are not acquired during the flyback portion of the gradient shape. Ramped gradients during readout move through *k*-space at the maximum possible speed, but produce irregular and unpredictable spacing of samples within *k*-space, exacerbating odd–even echo inconsistencies and complicating reconstruction. Irregularly sampled *k*-space data may be regridded [170, 273] based on a reference *k*-space trajectory measurement [95]. EPSI acquisition can also be accelerated using compressed sensing, by sparsely sampling *k*-space and frequency information by applying blipped phase-encode gradients during the EPSI echo train [79, 158, 159, 178, 179, 181], or using parallel imaging [276]. Example EPSI pulse sequences are shown below, with symmetric fully sampled readout (Fig. 7), and with flyback pseudo-randomly under-sampled readout (Fig. 8). A simplified model *k*-space trajectory, illustrating jumps between *k*-space lines, is shown in Fig. 9.

### Spiral readout methods

**Fig. 10:**
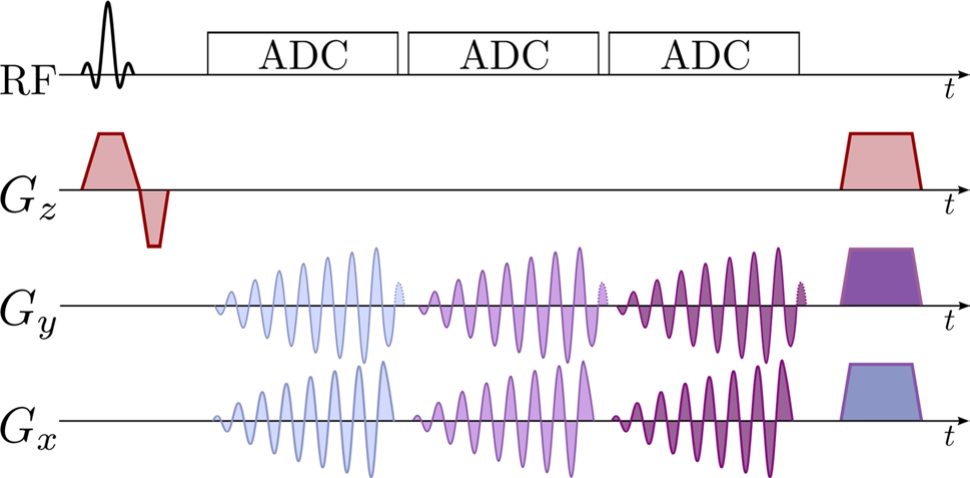
Pulse sequence using multi-echo spiral *k*-space trajectory, with slice-selective excitation followed by a series of gradient spirals during acquisition, and finally spoiler gradients

**Fig. 11:**
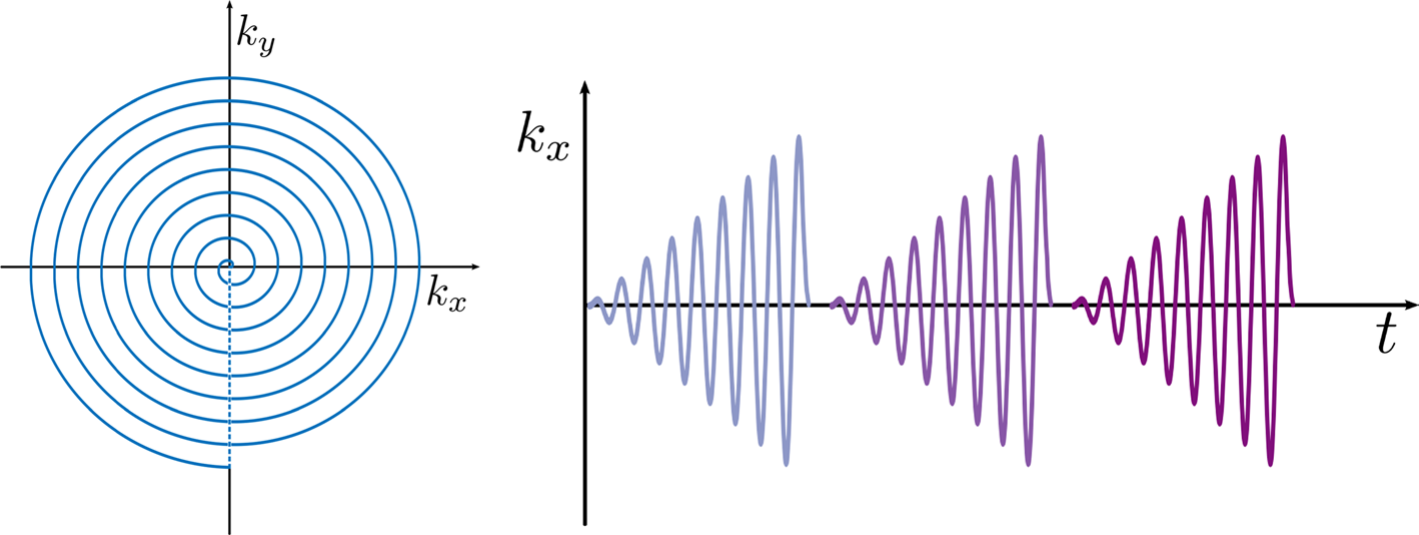
Spiral *k*-space trajectory, left: *k*_*x*_–*k*_*y*_ projection and right: *k*_*x*_–*t* projection

Spiral sequences use continuously varying gradients during readout to move in a spiral trajectory from the center to the periphery of 2D *k*-space [277]. A third spatial dimension may be measured by acquiring multiple slices separately [70, 278], or by applying phase encoding before readout or between multiple spiral readouts [168, 199]. Example pulse sequences using spiral readouts are shown in Fig. 1, with a single spiral per excitation, and in Fig. 10, with multiple spiral gradient echoes per excitation. Projections of *k*–*t* space illustrating the trajectory of a multi-echo spiral readout are shown in Fig. 11.

**Fig. 12:**
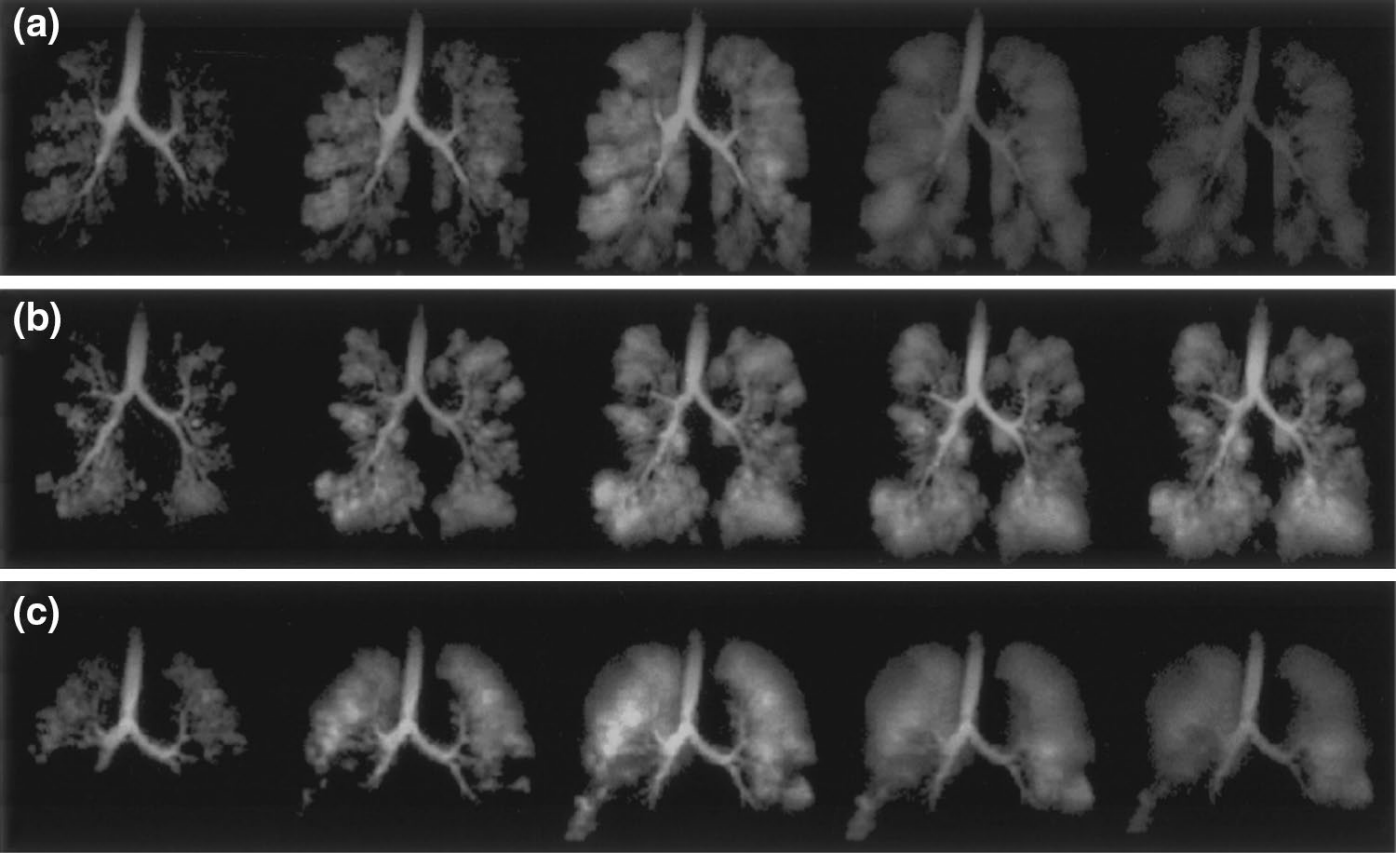
Dynamic spiral readout hyperpolarized ^3^He lung images of patients demonstrating variations in lung ventilation with **a** severe asthma, **b** cystic fibrosis, **c** emphysema secondary to α-1 antitrypsin deficiency. Reproduced with permission from Salerno et al. [11]

**Fig. 13:**
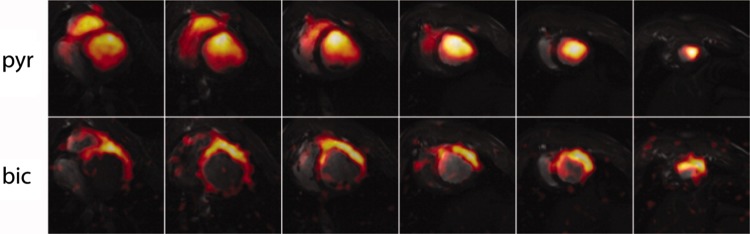
In vivo short-axis pig heart multi-slice dynamic cardiac-gated images of hyperpolarized ^13^C pyruvate (top row) and bicarbonate (bottom row) acquired with a spiral *k*-space trajectory after single-metabolite-resonance spectral-spatial RF excitations. Reproduced with permission from Lau et al. [70]

Because they sample the center of *k*-space first and after every excitation, spiral trajectories give good signal to noise ratio (SNR), can be used to implement self-navigating sequences [279], and can acquire with very short echo times. Spiral trajectories also sample *k*-space rapidly and efficiently [270], and can acquire one or more full 2D *k*-spaces in a single shot. These properties are beneficial for hyperpolarized imaging with non-renewable magnetization, particularly when acquiring dynamic image series [66, 280] (Figs. 12 and 13) or when the magnetization preparation unavoidably consumes all longitudinal magnetization. Spiral gradient trajectories are also relatively insensitive to motion and flow effects [278].

A disadvantage of spiral trajectories is that they are prone to *B*_0_ non-uniformity related distortions [131, 279], and thus require good shimming, more so at higher field strengths, but less so for X-nuclei with lower gyromagnetic ratios, and less apparently with lower image matrix sizes. More complicated reconstruction is also required due to the non-Cartesian *k*-space sampling [168–170]. Irregular *k*-space sampling is also difficult to combine with parallel imaging [279].

**Fig. 14:**
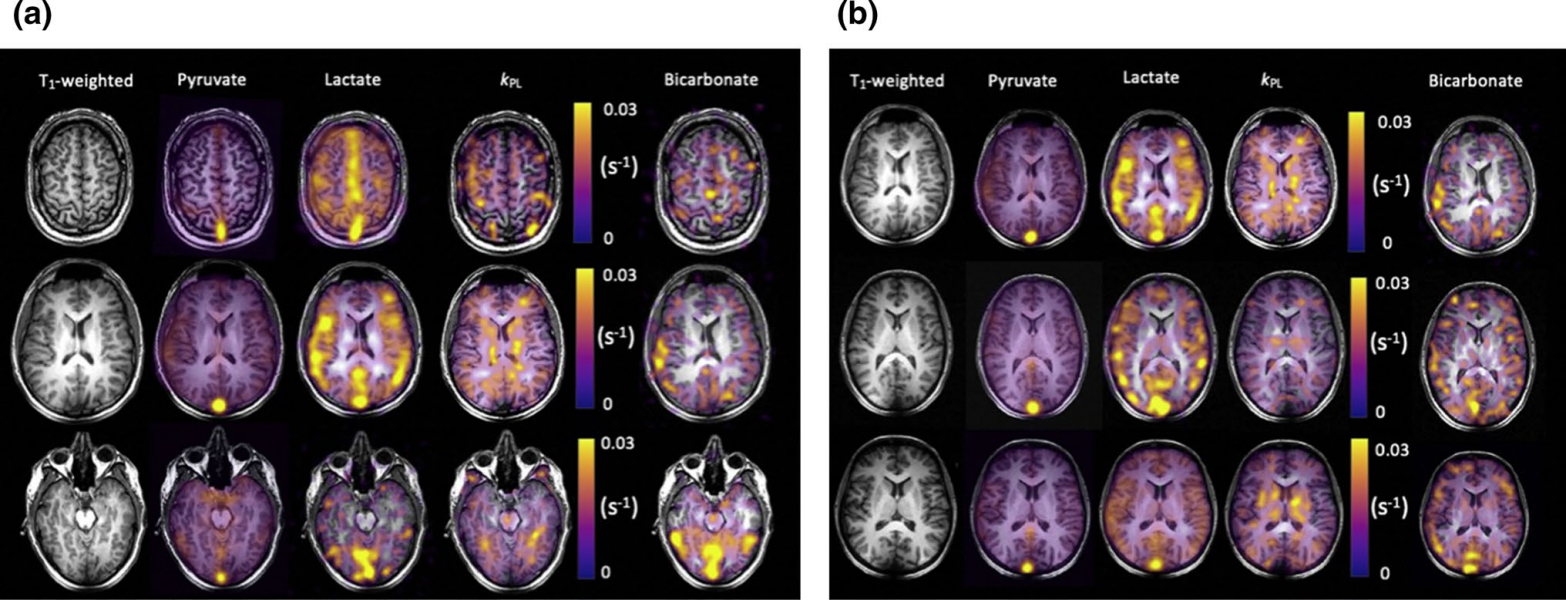
IDEAL spiral hyperpolarized ^13^C-labeled metabolite imaging in the healthy human brain in two subjects (**a**) and (**b**). Reprinted with permission from Grist et al. [111]

When spectral encoding during readout is not required, due to there being only a single measurable compound, e.g. with hyperpolarized gases (Fig. 12) or after a spectrally selective excitation [70] (Fig. 13), single-shot [277] or interleaved [11, 168, 278, 280] spiral readouts may be used. For imaging with spectroscopic acquisition (Fig. 14), the readout is repeated with multiple echo times, using a multiple-echo flyback trajectory or with shifting delays after excitations [125, 131, 168]. These echoes may be uniformly spaced and interpreted as a model-free multi-echo time series, or their timing may be varied to optimize separation of spectral peaks at expected chemical shifts using a sparse spectral sampling scheme [131].

Robustness to flow and motion makes cardiac imaging an important application of spiral readout sequences [70, 278, 281]. Acquisition with very short echo times is beneficial for hyperpolarized gas imaging, which can have very short *T*_2_* and strong diffusion effects [11, 280].

### Balanced steady-state free precession

Balanced steady-state free precession (bSSFP, also known as TrueFISP, balanced FFE, or FIESTA) is fast, magnetization efficient, and can provide a high SNR [282, 283]. The bSSFP sequence uses a spin echo train with repetition time (TR) much less than the transverse (*T*_2_) and longitudinal (*T*_1_) relaxation times, and balanced gradients with zeroth gradient moment equal to zero. For thermally polarized samples, a steady-state transverse magnetization is maintained [200] following an initial transient [282]. For hyperpolarized ^129^Xe [62, 284] or, e.g. ^13^C in liquid, a pseudo steady-state is reached in which the hyperpolarized magnetization is gradually consumed [150] after inhalation or administration. In either case, an initial half-alpha half-TR preparation RF pulse [285] can help reach the (pseudo) steady-state much faster [150, 283].

Balanced gradients and refocusing each TR lead to characteristic and well-defined banding artifacts for off resonant spins [286] that can also cause considerable signal and effective slice thickness variations across the imaging slice for spins near the stopbands [287]. For non-spectroscopic imaging, the central passband of this frequency response is centered on the RF transmission (*B*_1_) frequency by alternating the phase of the excitation pulses [283].

**Fig. 15:**
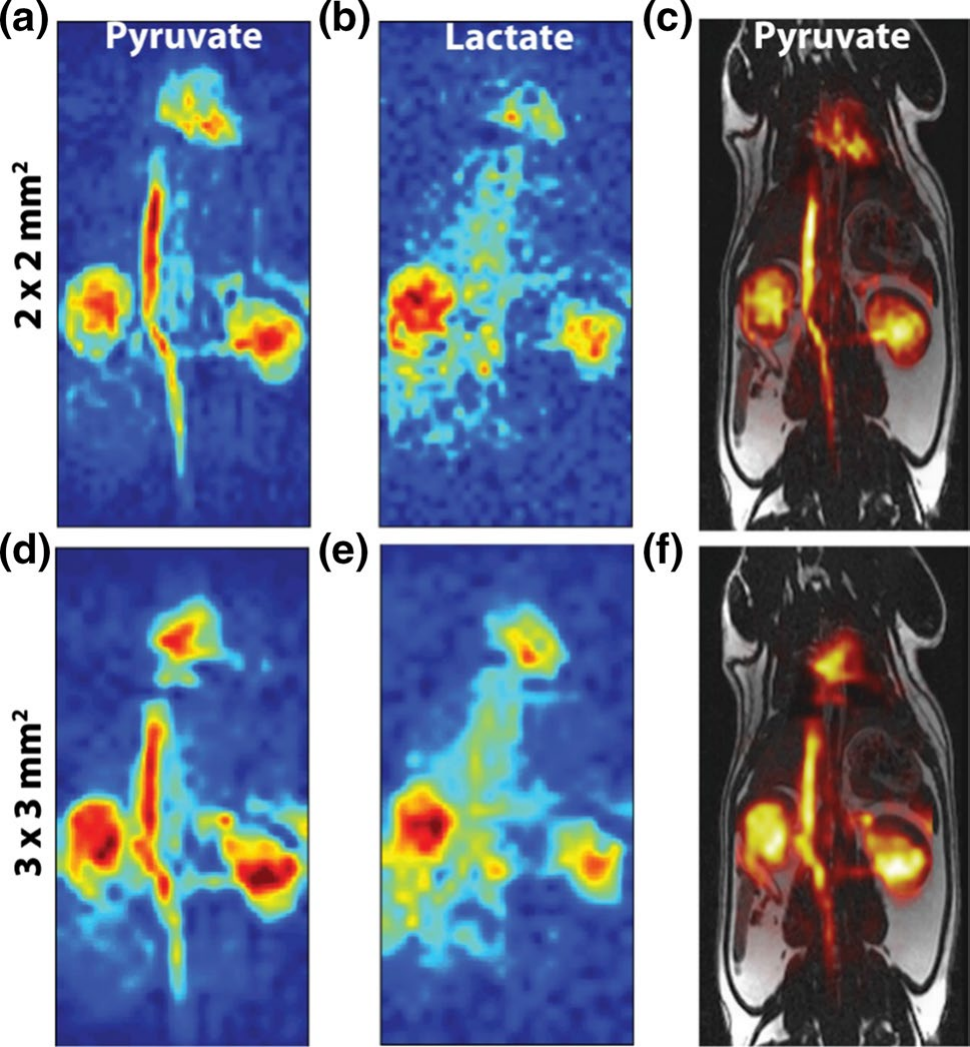
In vivo ^13^C rat abdomen bSSFP projection images of pyruvate and lactate acquired 20 s after start of injection of hyperpolarized pyruvate, with spectral suppression of alanine and pyruvate hydrate resonances before slab-selective excitation and one gradient echo per α RF pulse, alternating between metabolite center frequencies. **a** Pyruvate and **b** lactate at 2 × 2 mm^2^ acquired in-plane resolution. **d** Pyruvate and **e** lactate at 3 × 3 mm^2^ acquired in-plane resolution. Pyruvate at **c** 2 × 2 mm^2^ and **f** 3 × 3 mm^2^ acquired in-plane resolutions. Reprinted with permission from Milshteyn et al. [290]

For imaging of multiple chemical shifts, careful consideration of sequence timing is required. Bloch simulations [233] can be employed to determine TRs that place the hyperpolarized resonances of interest in the passbands of the bSSFP frequency response and undesired resonances in the stopbands [134, 282, 283, 288]. Various approaches based on combining acquisitions of different constant RF pulse phase increments [289] or varying the TR have been developed to alleviate or emphasize this frequency response, as desired. Multiple resonances can then be measured individually and sequentially using an interleaved, single readout [288] (Fig. 15), or concurrently using a sparse multi-echo, multi-readout approach [202]. Spectrally selective RF pulses are generally not used as refocusing pulses within the bSSFP due to their long pulse durations [288], but they have been used as a preparation [290].

High-resolution, temporally interleaved 3D time course metabolic imaging of co-polarized pyruvate and urea has been performed at 14 T with bSSFP, with metabolite separation enhanced by the use of optimized spectrally selective pulses [288, 291]. The same approach is feasible at 3 T, but saturation pulses prior to each image are required in order to suppress the alanine and pyruvate-hydrate resonances [290]. However, the use of such pulses reduces the rate at which metabolic images can be recorded.

**Fig. 16:**
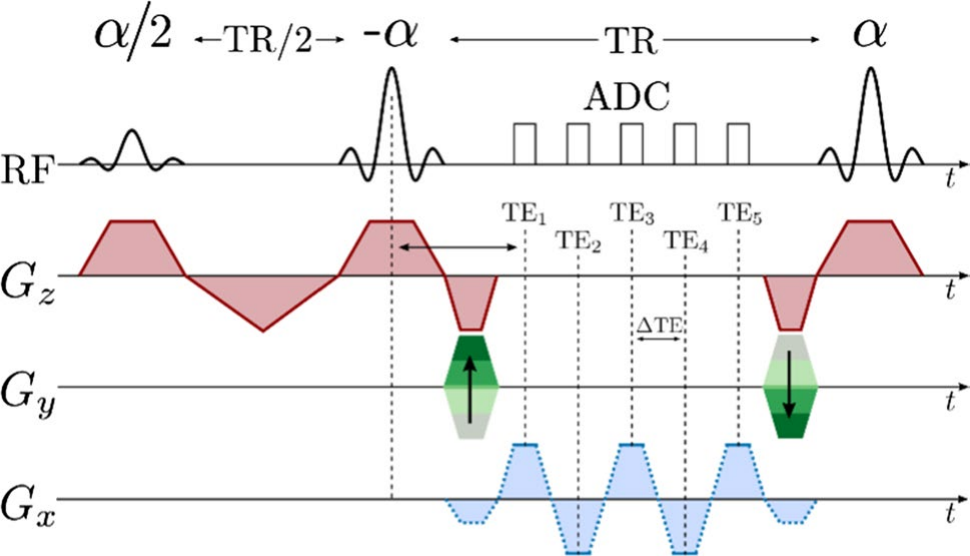
Balanced steady-state free-precession pulse sequence with multiple symmetric bipolar gradient echoes and signal acquisitions per RF pulse. Spectral information is under-sampled due to the small number of acquired echo times, and is, therefore, reconstructed with an IDEAL approach. The initial α/2 pulse brings the magnetization quickly into a pseudo-steady state. All zeroth-order gradient moments equal zero

**Fig. 17:**
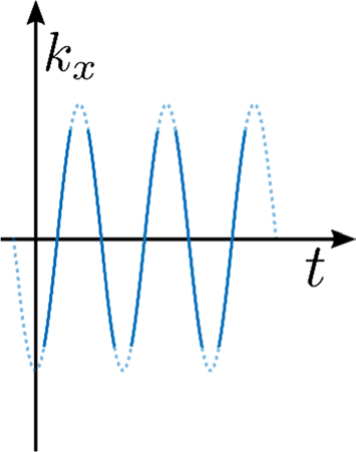
Symmetric readout bSSFP sequence *k*_*x*_ vs. time trajectory. Signal is read out during constant plateau portions of bipolar readout gradients

Sparse multi-echo bSSFP may be used to separate multiple chemical shift signals in a single measurement by acquiring a short symmetric train of gradient echoes after each RF pulse, which sample the envelope of the spin echoes [292, 293]. Based on the principle that each distinct resonance frequency will have a distinct phase evolution, iterative least-squares (IDEAL) reconstruction can be used to reconstruct separate images of multiple chemical shifts [135], such as hyperpolarized ^13^C pyruvate, alanine, and lactate [202]. This approach has been extended with bipolar gradient correction [164], and has been applied in tumor-bearing rats on a clinical 3 T scanner [294]. An example multi-echo bSSFP pulse sequence with multiple gradient echoes per RF excitation is shown in Fig. 16, and an illustration of the *k*_*x*_ trajectory is shown in Fig. 17.

Further applications of bSSFP in hyperpolarized imaging include non-frequency selective hyperpolarized gas lung imaging in both 2D [284] and 3D [295, 296], single [150] and multi-compound [297] hyperpolarized ^13^C angiography, rapid imaging of the heart [298], and numerous metabolic imaging approaches [108, 134, 202, 288].

### Spectral-spatial excitation

Spectral-spatial (SPSP) radiofrequency (RF) pulses are simultaneously selective in both frequency and space. They were originally designed for separating fat and water resonances [299], but have since proven particularly useful for imaging hyperpolarized ^13^C-labeled compounds and their metabolites [72]. In this application, SPSP pulses selectively excite individual metabolites, in contrast to multi-echo and free induction decay (FID) sequences that extract spectral information from a time-series of signal measurements. Single resonance SPSP pulses allow much faster encoding of *k*-space, because spectral encoding during readout is not required.

SPSP pulses consist of a series of shaped RF sub-pulses that are transmitted during a periodic oscillating magnetic field gradient. As a rough approximation, the shape of each sub-pulse determines the spatial profile, while the envelope of sub-pulses determines the spectral profile [72]. Important design parameters for SPSP pulses are the duration of sub-lobes (determining the width of the spectral stop-band), the shape of the envelope (determining the width of the spectral passband), and the total length of the pulse (determining the width of the transition between passband and stopband). Several strategies have been proposed for optimized design of SPSP pulses [139, 140, 300], including a free online software-package that computes RF and gradient pulses for a given specification on spectral and spatial excitation range [301].

**Fig. 18:**
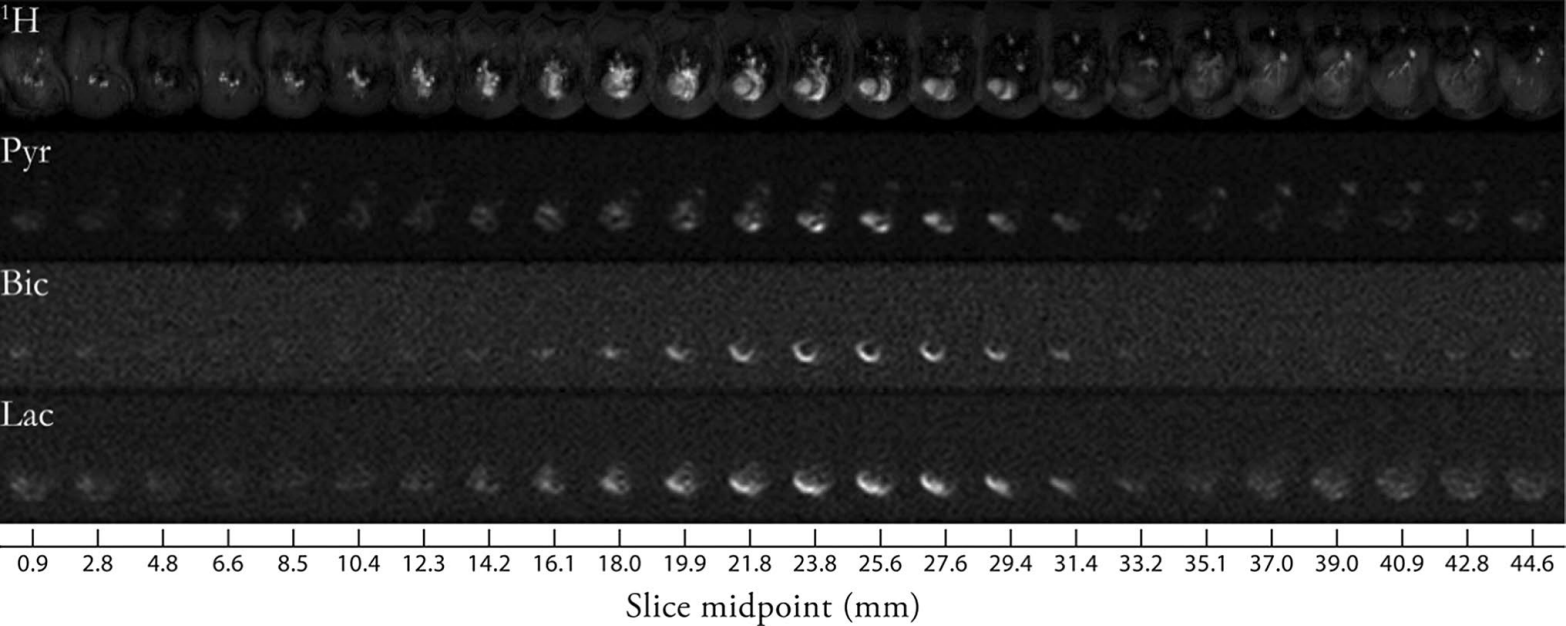
^1^H anatomical (top) and ^13^C-pyruvate, bicarbonate, and lactate images in in vivo rat heart, acquired with a series of single-metabolite-frequency spectral-spatial slab excitations and 3D EPI readouts. Reproduced from Miller et al. [302]

SPSP pulses can be designed to excite either a single resonance [139] or multiple resonances (multi-band) [140]. For the case of a single resonance excitation, the SPSP pulse is usually combined with a fast imaging readout in two or three spatial dimensions, such as echo-planar imaging [85, 141, 302] (Fig. 18) or single-shot spirals [66, 199, 303]. By shifting the excitation frequency of the SPSP pulse, different metabolites may be consecutively encoded.

Using multi-band SPSP excitation pulses, multiple selected metabolites can be excited with optimized individual flip angles, to more efficiently use the hyperpolarized magnetization [140, 301, 304]. In this case, chemical shift imaging techniques are required to separate the individual metabolite signals. A hybrid multi-echo spectral-spatial EPI sequence has been shown to achieve improved tolerance to off-resonance effects [85].

Due to their efficient and fast encoding, SPSP excitation acquisition schemes are advantageous when a high spatial resolution is required or when the acquisition needs to be fast because of motion, such as in the heart. Numerous studies have applied SPSP pulses for metabolic imaging in animals, in tumors, as well as in the heart, liver, and kidney (Table 1). Recently, they have also been used to study pyruvate metabolism in human subjects, focusing on healthy brain [305], healthy heart [76], and prostate and brain cancer [80].

**Table 1:** In vivo imaging strategies for ^13^C-labeled dDNP-polarized hyperpolarized probes, sequence details, respective applications and probed species

Strategy	Additional specifics of acquisition strategy	HP-probe	Application	Species
FIDCSI	3-Dimensional [373] Compressed Sensing [374] ECG-gated and respiratory-gated [241] Parallel imaging [83] Variable flip angle [375]	[1-^13^C]pyruvate	Tumor Brain Heart Inflammation Kidney Lung Obesity *T*_2_-mapping	M [65], R [343], D [376] M [377], R [368] R [378], P [167] R [375] R [379], P [380] R [381] M [382] R [383]
		[1-^13^C]acetoacetate	Redox	R [25]
		[1-^13^C]bicarbonate	pH	M [5]
		[1-^13^C]dehydroascorbate	Redox	M, R [373]
		[1-^13^C, U-^2^H]ethanol	Liver	M [370]
		[1-^13^C]ethyl pyruvate	Brain	R [368]
		[1,3‐^13^C_2_]ethyl acetoacetate	Tumor	R [354]
		[1,4-^13^C_2_]fumarate	Necrosis	M [6], R [358]
		[U-^13^C, U-^2^H]glucose	Tumor	M [353]
		[1-^13^C]α-ketobutyrate	Heart, kidney	R [363]
		[1‐^13^C]2-ketoisocaproate	Tumor	M [357]
		[2-^13^C]pyruvate	Brain	R [367]
		[1,2-^13^C]pyruvate	Heart, kidney	R [363]
		[1,5-^13^C_2_]zymonic acid	pH	R [50]
SPCSI	3-Dimensional [361] Bipolar flow-sensitive gradients [384] ECG-gated and respiratory-gated [385] Compressed Sensing [372] Multi-echo [344] Multi-echo-SPARSE [125, 131] Multi-slice [125] Variable flip angle [344, 386]	[1-^13^C]pyruvate	Tumor Brain Heart Kidney Liver Pregnancy	R [125] R [54], P [387], H [111] P [386] R [344], R [388] R [389]
		[1-^13^C]acetate	Kidney	R [366]
		[1-^13^C]acetoacetate	Heart	R [372]
		[2-^13^C]dihydroxyacetone	Liver	R [372]
		[1-^13^C]ethyl pyruvate	Brain	P [242]
		[1,4-^13^C_2_]fumarate	Necrosis Kidney	R [359] R [360]
		[1-^13^C]ketoisocaproate	Brain	R [369]
		[1-^13^C]lactate	Heart	R [361]
		[^13^C]urea	Perfusion	R [350]
		[^13^C,^15^N_2_]urea	Perfusion	R [359]
EPSI	3-Dimensional [390] Absorptive mode [49] Bipolar flow-sensitive gradients [391] Compressed sensing [158, 159] Double spin echo [128] Flyback readout [128] Parallel imaging [84] Symmetric readout [273, 392] WALTZ16-decoupling [228] Variable flip angle [82]	[1-^13^C]pyruvate	Tumor Brain Kidney Liver	M [82], R [393], D [273], H [78, 79, 272, 305] M [49] R [128] R [128]
		[1-^13^C]bicarbonate	pH	M [394]
		[1-^13^C]dehydroascorbate	Kidney	M [392]
		[2-^13^C]fructose	Tumor	M [355]
		[1-^13^C]lactate	Kidney, liver	R [371]
		[^13^C]urea	Perfusion	M [346]
SSFP	3-Dimensional [365] Bolus tracking [348] Compressed sensing [307] Multi-echo-SPARSE [204, 292] Multi-slice [346] Spectral suppression [290] Variable flip angle [290]	[1-^13^C]pyruvate	Tumor Heart Kidney	M [307] P [298] P [292]
		[1-^13^C, U–H_2_]tert-butanol	Perfusion	M [297], R [345]
		HP001	Perfusion	M [346], R [348]
		[1-^13^C]lactate	*T*_*2*_-mapping	M [307]
		[2-^13^C]pyruvate	*T*_*2*_-mapping	M [307]
		[^13^C]urea	Angiography Kidney Perfusion *T*_2_-mapping	R [34, 395] R [396] M [297] R [107]
		[^13^C,^15^N_2_]urea	Kidney Perfusion *T*_2_-mapping	R [364], P [365] P [349] R [107]
SPSP	3-Dimensional [139] *B*_1_-mapping [73] Bolus-tracking [397] Compressed sensing [235] Double spin echo [140] ECG-gated [70] FID-CSI [398] Unpaired adiabatic pulses [199] INEPT [216] *k*–*t* Principal component analysis [74] Multi-band radial frequency encoding [399] Multi-echo-SPARSE SPCSI [362] Mult-islice [70] Parallel imaging [86] Saturation recovery [66] Selective non-excitation of pyruvate [400] Multi-echo SPCSI [72] Steady state free precession [291] Flyback echo planar spectroscopic imaging [140] Symmetric echo planar imaging [95] Variable flip angle [140]	[1-^13^C]pyruvate	Tumor Brain Diffusion Heart Obesity	M [139, 140], R [66], H [80] NHP [401], H [119] M [269] R [402], P [70, 72], H [76] R [403]
		[1-^13^C]acetate	Heart	R [362]
		[1-^13^C]bicarbonate	pH	R [404]
		[2-13C]dihydroxyacetone	Kidney, liver	R [301]
		[1-^13^C]glycerolcarbonate	pH	M [398]
		[1-^13^C]α-ketoglutarate	Tumor	R [356]
		HP001	Perfusion	M [347]
		[2-^13^C]pyruvate	Liver *T*_2_-mapping	R [405]
		[^13^C]urea	Angiography	M [288]
		[^13^C,^15^N_2_]urea		

*M* mouse, *R* rat, *P* pig, *D* dog, *NHP* non-human primate, *H* human

### Relaxometry

Relaxometry measurements of hyperpolarized agents can determine their longitudinal (*R*_1_ or *T*_1_) and transverse (*R*_2_ or *T*_2_ and *R*_2_* or *T*_2_*) magnetization relaxation rates or times. Several ^13^C labeled compounds’ *T*_1_ and *T*_2_ times, and ^129^Xe gas or in solution *T*_1_ times, are on the order of tens of seconds in vivo, allowing a series of excitations to sample the *T*_1_ decay or a series of echoes to sample the *T*_2_ decay.

The transverse magnetization relaxation from atomic and molecular interactions (*R*_2_ or *T*_2_) can be measured using a multi-spin echo experiment with a train of refocusing pulses [198, 306, 307] after a single excitation. With hyperpolarized compounds, this is often limited to slice selective [138, 308] or voxel localized [309] experiments. When imaging *T*_2_, the results are dependent on echo spacing [310, 311], *B*_1_ calibration [312], and resonance-offsets [313], and are described as an effective *T*_2_ [107]. Transverse relaxation can be difficult to assess in hyperpolarized gas imaging due to their relatively high diffusion rates, which confound the relaxation signal change [145].

**Fig. 19:**
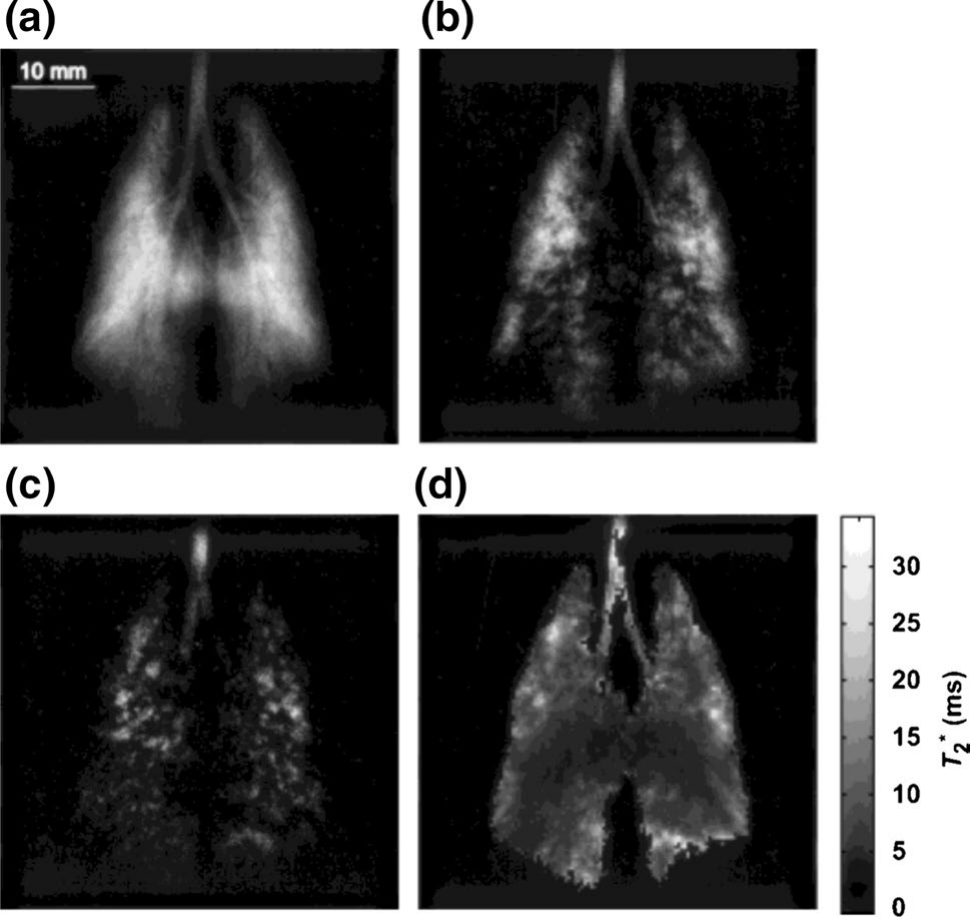
*T*_2_-weighted non-slice-selective images of ^3^He in guinea pig lung at **a** TE = 0.26 ms, **b** TE = 7 ms, and **c** TE = 16 ms. The calculated *T*_2_* map is shown in (**d**). Reprinted with permission from Chen et al. [123]

The transverse relaxation including static field (*B*_0_) inhomogeneity effects (*R*_2_* or *T*_2_*) can be derived from FID measurements [123, 147, 314]. In cases with minimal variation across the excited slice or volume, a simple non-imaging FID can be fit. Alternatively, a FID-CSI reconstruction can be used to spatially localize spectral peaks, from which a peak width can be fit, with sensitivity only to local *B*_0_ variations from susceptibility variations. For very short *T*_2_* compounds, frequency-encoding with center-out radial [123] (Fig. 19) or spiral trajectories can be used to minimize the shortest echo times. Alternatively, multi-gradient echo images can be fit for *T*_2_* [315].

Longitudinal relaxation (*R*_1_ or *T*_1_) can be determined from the signal magnitude in a series of FID or image acquisitions [34, 147] when no substantial addition, removal, or conversion between hyperpolarized compounds in the volume of interest is expected. Chemical reactions or exchange (e.g. pyruvate to lactate conversion) will alter the affected compounds’ signal magnitudes over time, but these processes can also be fit as part of a multi-compartment kinetic modeling experiment [65, 120, 316] that includes one or more *T*_*1*_ relaxation parameters. Perfusion or flow into or out of the volume of interest, and metabolic conversion into compounds that are not measured in the experiment, are more challenging; additional free parameters may be added to a kinetic model to account for them, but extra degrees of freedom can lead to noisier fit results or even physically or physiologically implausible results. The magnetization lost to previous excitation RF pulses [34] and the differing signal after varying excitation angle [181] must also be accounted for.

## In vivo imaging applications of hyperpolarized substances

Hyperpolarization allows for functional and metabolic in vivo imaging relying on a great variety of different probes. Noble gases as well as small inorganic and organic molecules bearing half-integer spin nuclei are commonly polarized with SEOP, PHIP and dDNP. This chapter summarizes molecules polarized with these techniques and describes their in vivo applications and the respective acquisition strategies. In Table 1, five of the most prominent in vivo acquisition strategies and in vivo applications of different metabolic probes that are labeled with ^13^C and polarized with dDNP are listed.

### Spin exchange optical pumping (SEOP)

Noble gases are exclusively polarized by SEOP and are used to characterize gas-filled spaces such as the respiratory tract and the lung [317]. In its early stages, the field of hyperpolarized gas MRI mainly focused on the assessment of lung ventilation and physiological parameters such as partial oxygen pressure [318, 319]. These techniques have been applied to asthma, apnea, cystic fibrosis, emphysema, chronic obstructive lung pulmonary disease, and smoking-related aberrant changes of the lung [320, 321]. Analyzed model systems included small animals (mouse, rat, guinea pig), rabbits, and dogs. Additionally, because ^3^He and ^29^Xe are chemically inert and FDA approved, children and adult patients were also examined [322, 323]. When encapsulated in microbubbles [324], microspheres [147], or as lipid emulsions [106], hyperpolarized noble gases can be delivered to the blood stream at fairly high concentrations and polarization levels, which allows for angiography, as well as brain and heart imaging. Although sparsely applied, hyperpolarized ^83^Kr has been shown to serve as imaging marker for lung emphysema, based on disease-related surface mediated quadrupolar *T*_1_ relaxation [325].

The most important in vivo acquisition strategies for hyperpolarized noble gases are based on spoiled gradient echoes [193], gradient recalled echoes (GRASS), echo planar (diffusion-weighted) imaging [146], and bSSFP [195]. FID-CSI has also been used to image blood-dissolved ^129^Xe in brain [326], heart, kidney, and lung [327].

### Parahydrogen induced polarization (PHIP)

In contrast to noble gases, small inorganic or organic molecules can be polarized by brute force, PHIP, SABRE, and dDNP. However, brute force and SABRE polarized molecules have so far not been used for in vivo MRI. PHIP, based on chemical addition of parahydrogen, relies on unsaturated precursor molecules, which limits the available probes and applications for this hyperpolarization technique. Several molecules such as [1-^13^C,D_8_]bis-1,1-(hydroxymethyl)-cyclopropane (HP001) [150], [1-^13^C]maleic acid dimethyl ester [149], and [1-^13^C,2,3,3D_3_]2-hydroxyethylpropionate [328–330] were developed for ^13^C-angiography in rats, rabbits, and pigs and measured with bSSFP-based acquisition strategies. Probes like [1-^13^C]succinate and its ethyl ester have, respectively, been applied in malignant brain [331] and kidneys [332] of rodents, but as yet without imaging of their metabolic products. Recently, hyperpolarized [1-^13^C]pyruvate was produced with PHIP-SAH (side arm hydrogenation) for the first time enabling PHIP-based in vivo metabolic chemical shift imaging [333].

### Dissolution dynamic nuclear polarization (dDNP)

dDNP is the most applied hyperpolarization technique for in vivo MRI, mainly using ^13^C-labeled molecules, but is also applied to other spin-1/2 nuclei including ^1^H, ^6^Li, ^15^N, and ^29^Si. Hyperpolarized ^29^Si-nanoparticles were developed for tumor perfusion imaging in mice using fast spin echo imaging [334], while spatially localized hyperpolarized ^6^-Li distribution was shown in rat brain using gradient recalled echo methods [28]. Hyperpolarized water served as an angiographic probe or as a heart and kidney perfusion marker in preclinical models of rats [335] and pigs [336, 337], measured with gradient echo or bSSFP methods, respectively. With regard to in vivo ^15^N-applications, so far only α-trideuteromethyl[^15^N]glutamine, exhibiting a long *T*_1_ for perfusion mapping with spiral sequences in rodents, has been demonstrated [338].

^13^C is the NMR active surrogate of NMR inactive ^12^C, which is one of the most important atomic components of metabolites in living species. The spin-1/2 nucleus has a fairly high gyromagnetic ratio, yields high polarization levels, and has *T*_1_ values of several tens of seconds. In addition, the availability of commercial preclinical [2] and clinical DNP polarizers [339] boosted the development of a variety of hyperpolarizable ^13^C-labeled probes during the last two decades, allowing for real-time metabolic imaging in vivo. An overview of the most important in vivo imaging strategies including sequence details, the hyperpolarized ^13^C-labeled molecules, and respective in vivo applications is given in Table 1.

The most extensively DNP-polarized molecule is [1-^13^C]-pyruvate. Its ^12^C-equivalent is the end product of glycolysis, which is the key metabolic pathway to generate energy and CO_2_ via oxidative phosphorylation. Two alternative metabolic pathways of pyruvate are mediated by alanine-aminotransferase and pyruvate dehydrogenase, which produce alanine and CO_2_, respectively. In several disease states, the pyruvate metabolism has shown to be aberrant. As prominent example, many tumors show increased consumption of glucose, while switching from oxidative phosphorylation to aerobic glycolysis producing lactate, even under normoxic conditions. This is known as the Warburg effect [340, 341]. Besides this biological importance, [1-^13^C]-pyruvate reaches polarization levels up to 70% [342], and has one of the longest *T*_1_ values for ^13^C-labeled molecules, which is one main reason why almost every ^13^C imaging strategy was developed with it. A summary of applications, with representative references using ^13^C-pyruvate for in vivo imaging of healthy and diseased states, is given in Table 1.

Five of the most important ^13^C-acquisition strategies are based on FID-CSI [65, 343], spiral CSI (SPCSI), [125, 344], bSSFP [292, 297], EPSI [128], and SPSP [140]. Applications of hyperpolarized pyruvate are very broad, including solid tumor imaging, metabolic imaging of healthy or diseased states of the brain, heart, lung, or kidneys, and of obesity and pregnancy. Based on extensive work and optimization of the imaging protocols in preclinical models including mice (M), rats (R), dogs (D), pigs (P), and non-human primates (NHP), ^13^C-imaging is now being translated to patients. Examples of human (H) applications include imaging of the healthy heart [76], healthy brain [111, 119], prostate tumors [78, 79], as well as untreated [80, 272] and treated [305] brain tumors.

Hyperpolarized pyruvate can also be used as angiographic agent or perfusion marker. However, because it is metabolically active, metabolically inert compounds exhibiting fairly long *T*_1_ have been polarized with dDNP and suggested as alternative perfusion markers. Prominent examples include [1-^13^C]butanol [297, 345], HP001 [346–348], [^13^C]urea [34], and [^15^N–^13^C]urea [349], measured mainly with bSSFP and, when co-polarized with other markers, FID-CSI [50], SPCSI [350], or EPSI [346].

Another metabolic property of tumors resulting from the Warburg effect is the export of excess lactate to the extracellular space, which leads to an acidification of the tumor microenvironment. Extracellular pH can be probed by hyperpolarized pH sensors, whose NMR signals either exhibit a ratiometric ([^13^C]bicarbonate/[^13^C]CO_2_ pair [5]) pH-sensitivity or pH-dependent chemical shifts ([1,5-^13^C]zymonic acid [50, 351]). Because chemical shift based pH-sensors require high spectral resolution and exhibit chemical shifts that are not known prior to measurement, FID-CSI is often used as the imaging strategy [352].

Tumors do not necessarily exhibit the Warburg effect, but may instead show other metabolic abnormalities. Therefore, tumor markers other than [1-^13^C]pyruvate, such as uniformly ^13^C-labeled and deuterated glucose [353], [1,3-^13^C_2_]ethyl acetoacetate [354], [2-^13^C]fructose [355], [1-^13^C]α-ketoglutarate [356], and [1-^13^C]2-ketoisocaproate [357], could also be helpful for precise and non-invasive characterization of tissues.

[1,4-^13^C_2_]fumarate is a metabolic probe indicating cell death. In vivo, the molecule is converted to [1,4-^13^C_2_]malate by malate dehydrogenase when released from cells undergoing necrosis, such as after tumor therapy or acute kidney injury [6, 358–360].

Like tumors, other non-communicable diseases such as cardiovascular diseases and diabetes are imminent problems for humanity. [1-^13^C]lactate [361], [1-^13^C]acetate [362], and [^13^C]α-ketobutyrate [363] have been applied to probe abnormal changes in the heart, while diabetic changes in the kidney have been assessed with [^13^C,^15^N_2_]urea [364, 365]. In contrast, renal clearance as a physiological parameter was quantified with [^13^C]acetate [366] and gluconeogenesis, as an example of organ-specific metabolic processes, has been imaged with hyperpolarized [2-^13^C]dihydroxyacetone [301].

Finally, few probes other than [1-^13^C]pyruvate have been mentioned for imaging of brain and liver metabolism. For the brain, these are [2-^13^C]pyruvate [367], [1-^13^C]ethyl pyruvate [242, 368], and [1-^13^C]2-ketoisocaproate [369]. For the liver, uniformly deuterated [1-^13^C]ethanol [370] probing aldehyde dehydrogenase activity, and [1-^13^C]lactate [371] and [2-^13^C]dihydroxyacetone [372] to image liver metabolism have been demonstrated.

## Conclusion

Over the past 25 years, the field of hyperpolarized MRI has made tremendous progress, evolving from basic science to preclinical and clinical studies based on multiple interdependent advances in polarization technologies, probe chemistry, imaging hardware, and acquisition methods. The need to use the dramatically enhanced hyperpolarized magnetization most efficiently—within a few tens of seconds—and, at the same time, to separate more than one resonance during the image acquisition, has led to the development of novel imaging methods. Hyperpolarized gas applications for measurements of lung perfusion could be translated to humans early on, building upon standard proton imaging protocols. For hyperpolarized liquids, multiple acquisition strategies to achieve fast, robust, and efficient dynamic spectroscopic imaging in up to three spatial dimensions have been presented, including chemical shift imaging techniques, spectral-spatial excitation, balanced steady-state free precession, and spiral imaging.

While realizing that there are innumerable possibilities in pulse sequence design, we have attempted to summarize the most commonly used pulse sequence components and their combinations for spectral encoding, spatial encoding, and excitation and contrast generation that have so far been presented in the context of hyperpolarized MRI. For the scientist who tries to tailor a pulse sequence for his or her needs to image an individual hyperpolarized probe molecule for a certain application, this could provide helpful information.

However, besides optimizing a pulse sequence for sensitivity, robustness and reproducibility of quantification are equally important for preclinical and clinical applications. The effect of individual pulse sequence components and parameters on the quantification of results as imaging biomarkers, e.g. pyruvate-to-lactate conversion rates *k*_pl_, has been discussed recently [391]: A profound effect of sequence parameters on *k*_pl_ was found for the cases of magnetization spoiling by RF pulses, flow suppression by crusher gradients, and intrinsic image weightings due to relaxation. Given the large variety of pulse sequence components used in hyperpolarized MRI, a major challenge for this field will be the standardization of acquisition protocols and data analysis procedures for comparison of results across different sites which, is especially important for the validation of biomarkers in clinical studies. A consensus has to be found within the hyperpolarized MR community on which acquisition and analysis strategies should be used for which application. A standardized vendor-independent platform to share acquisition protocols as well as data analysis procedures for quantification of imaging measures such as *k*_pl_-maps would help to address this challenge.
